# Extended reality used in the treatment of phantom limb pain: a multicenter, double-blind, randomized controlled trial

**DOI:** 10.1097/j.pain.0000000000003384

**Published:** 2024-09-05

**Authors:** Eva Lendaro, Corry K. Van der Sluis, Liselotte Hermansson, Lina Bunketorp-Käll, Helena Burger, Els Keesom, Cathrine Widehammar, Maria Munoz-Novoa, Brian E. McGuire, Paul O’Reilly, Eric J. Earley, Sonam Iqbal, Morten B. Kristoffersen, Anita Stockselius, Lena Gudmundson, Wendy Hill, Martin Diers, Kristi L. Turner, Thomas Weiss, Max Ortiz-Catalan

**Affiliations:** a Department of Electrical Engineering, Chalmers University of Technology, Goteborg, Sweden; b Department of Brain and Cognitive Sciences, McGovern Institute for Brain Research, Massachusetts Institute of Technology, Cambridge, MA, United States; c University of Groningen, University Medical Center Groningen, Department of Rehabilitation Medicine, Groningen, the Netherlands; d Department of Prosthetics and Orthotics, Faculty of Medicine and Health, Örebro University, Örebro, Sweden; e Faculty of Medicine and Health, University Health Care Research Centre, Örebro University, Örebro, Sweden; f Department of Health and Rehabilitation, Institute of Neuroscience and Physiology, Sahlgrenska Academy, University of Gothenburg, Gothenburg, Sweden; g Centre for Advanced Reconstruction of Extremities, Sahlgrenska University Hospital, Gothenburg, Sweden; h University Rehabilitation Institute, Ljubljana, Slovenia; i Faculty of Medicine, University of Ljubljana, Ljubljana, Slovenia; j Department of Pediatric Rehabilitation, Treant Hospitals, the Netherlands; k Integrum AB, Mölndal, Sweden; l Center for Bionics and Pain Research, Mölndal, Sweden; m School of Psychology & Centre for Pain Research, University of Galway, Galway, Ireland; n Department of Orthopedics, University of Colorado Anschutz Medical Campus, Aurora, CO, United States; o Bone-Anchored Limb Research Group, University of Colorado Anschutz Medical Campus, Aurora, CO, United States; p Department of Orthopaedics, Institute of Clinical Sciences, Sahlgrenska Academy, University of Gothenburg, Sweden; q Rehabcenter Sfären, Bräcke Diakoni, Stockholm, Sweden; r Institute of Biomedical Engineering, University of New Brunswick, Fredericton, NB, Canada; s Department of Psychosomatic Medicine and Psychotherapy, LWL University Hospital, Ruhr Universität Bochum, Germany; t Center for Bionic Medicine, Shirley Ryan Ability Lab, Chicago, IL, United States; u Department of Clinical Psychology, Friedrich Schiller University Jena, Jena, Germany; v Bionics Institute, Melbourne, Australia; w University of Melbourne, Melbourne, Australia

**Keywords:** Pain, Phantom limb, Amputation, Virtual reality exposure therapy, Augmented reality, Rehabilitation, Pain management

## Abstract

Phantom limb pain (PLP) represents a significant challenge after amputation. This study investigated the use of phantom motor execution (PME) and phantom motor imagery (PMI) facilitated by extended reality (XR) for the treatment of PLP. Both treatments used XR, but PME involved overt execution of phantom movements, relying on the decoding of motor intent using machine learning to enable real-time control in XR. In contrast, PMI involved mental rehearsal of phantom movements guided by XR. The study hypothesized that PME would be superior to PMI. A multicenter, double-blind, randomized controlled trial was conducted in 9 outpatient clinics across 7 countries. Eighty-one participants with PLP were randomly assigned to PME or PMI training. The primary outcome was the change in PLP, measured by the Pain Rating Index, from baseline to treatment cessation. Secondary outcomes included various aspects related to PLP, such as the rate of clinically meaningful reduction in pain (CMRP; >50% pain decrease). No evidence was found for superiority of overt execution (PME) over imagery (PMI) using XR. PLP decreased by 64.5% and 68.2% in PME and PMI groups, respectively. Thirty-seven PME participants (71%) and 19 PMI participants (68%) experienced CMRP. Positive changes were recorded in all other outcomes, without group differences. Pain reduction for PME was larger than previously reported. Despite our initial hypothesis not being confirmed, PME and PMI, aided by XR, are likely to offer meaningful PLP relief to most patients. These findings merit consideration of these therapies as viable treatment options and alternatives to pharmacological treatments.

## Abstract 2

Supplemental Digital Content is Available in the Text.

The use of phantom motor execution and phantom motor imagery aided by extended reality substantially alleviates phantom limb pain and its associated comorbidities.

## 1 Introduction

Phantom limb pain (PLP), a neuropathic pain perceived in the absent limb, is a frequent result of amputation. This condition's clinical prevalence is around 60%, and escalates to a lifetime prevalence of 76% to 87%.^6,33^ Despite being frequent, effective treatments are elusive, with limited and possibly biased evidence supporting long-term efficacy of both pharmacological and nonpharmacological interventions^1,3,9,22^

In recent years, extended reality (XR)—encompassing augmented reality (AR) and virtual reality (VR)—has garnered interest as a potential tool for treating chronic pain.^10^ The typical methodology for reducing chronic pain through XR treatments involves strategies such as self-hypnosis, guided imagery, biofeedback or cognitive behavioral therapy.^20^ Significantly, a VR device has recently been approved by the U.S. Food and Drug Administration for the treatment of chronic back pain.^8^ Applied to PLP, systematic reviews have highlighted several studies reporting significant pain reduction,^34^ although high-quality evidence from randomized controlled trials (RCTs) is still lacking.

Here, we report the first international, double-blind RCT comparing the effects of 2 distinct XR-based treatments for chronic PLP (persisting beyond 3 months). In the experimental group, we used myoelectric pattern recognition innovatively to infer the intention to execute movements of the absent limb by decoding electrical signals generated from the contraction of residual muscles. This technique builds on the fact that phantom movements can activate the muscles still present in the amputated limb. For instance, muscle activity in limbs amputated above the elbow or above the knee can still produce signals discernible enough to decode hand and foot movements, respectively.^7,13^ Ortiz-Catalan et al. used this approach showing clinically meaningful alleviation of PLP (∼50%) in a single-arm trial.^27^ Although this treatment is now used clinically around the world, no RCT has been conducted. In this study, we used phantom motor imagery (PMI) as an alternative to phantom motor execution (PME) in an RCT. In the active control group (PMI), we examined the use of XR in the context of motor imagery, where users synchronize their imagination of a movement with an autonomously moving XR limb. Myoelectrical signals were used to monitor that patients did not produce identifiable muscular contractions while imagining the movements.

Prior research has shown that overt execution of phantom movements, motor imagery, and action observation can reduce PLP.^4,15,19,27,28^ Executed phantom movements are associated with kinetic sensations analogous to those felt in the limb before amputation^31^ but require more effort than imagined movements^30^ and are thought to activate the sensorimotor system in ways that are similar to movements of the intact limb.^5^ A recent hypothesis for the origin of PLP suggests that pain relief depends on the engagement of motor and somatosensory circuitry, and that re-engaging them to a greater extent in a physiological way should lead to greater pain relief.^26^ Following this rationale, phantom motor execution (PME) was postulated as a superior mechanism in driving relief of PLP compared with phantom motor imagery (PMI), motivating the design of this study as a superiority trial.^26^ The present study aimed to compare the efficacy of PME against PMI using the same training protocol, device, and virtual environments.

## 2 Methods

### 2.1 Study design and participants

We conducted a multicenter, double-blind, superiority RCT at 9 outpatient clinics in 7 countries (Sweden, the Netherlands, Slovenia, Ireland, Germany, Canada, and the United States; Table S1, http://links.lww.com/PAIN/C120). Articles describing the trial protocol and statistical analysis plan were published at the early stages of the trial and before the beginning of data analysis, respectively.^17,18^ The trial adhered to the Good Clinical Practice and the Declaration of Helsinki principles, and its protocol was reviewed and approved by the local Ethics Committee of each partner clinic. The results are reported in accordance with the CONSORT (Consolidated Standards of Reporting Trials) guidelines.^32^

E.L. and M.O.C. vouch for the accuracy and completeness of this report and its fidelity to the study protocol. Potential participants were screened for eligibility by the participating centers. The study comprised a baseline assessment (visit 0), 15 treatment sessions, and 3 follow-up interviews at 1, 3, and 6 months posttreatment. Complete eligibility criteria and visit schedules are described in Table 1 and Table 2, respectively. All participants provided written informed consent before study-related procedures. Sex was self-reported as female or male.

**Table 1:** List of eligibility criteria.

Individuals must meet all the following inclusion criteria to be eligible to participate in the study	
1	The participants must be older than 18 y with chronic PLP
2	The participant must have signed a written informed consent
3	Participants must have chronic PLP, with at least 6 mo passed since amputation; participants with acute PLP are noneligible
4	In case of pharmacological treatments, the dosage must have been stable for the previous month
5	Any previous PLP treatments must have terminated at least 3 mo before entering the study
6	Any pain reduction potentially attributable to previous PLP treatments must have occurred at least 3 mo before entering the study
7	The patient participant should not have a significant cognitive impairment that prevents the patient from following instructions
8	Voluntary control over at least a portion of biceps and triceps muscles in case of upper limb amputation or quadriceps and hamstrings in case of lower limb amputation
9	Stable prosthetic situation (ie, satisfaction with the fitting of the prosthesis) or being a nonuser
10	No abundant soft tissue on the stump that prevents sufficient myoelectric signals from being recorded
11	No presence of pain >2 on Numerical Rating Scale (NRS) on contact with the skin or muscle contraction in the stump
12	The PLP must not be aggravated (NRS >4) by the execution or imagination of phantom movements
13	No condition associated with risk of poor protocol compliance
14	No injury, disease, or addiction that would render the individual unsuitable for the trial
15	Pain rating index (PRI) >0 as assessed in the Questionnaire for Phantom Limb Pain (Q-PLP) at visit 0

**Table 2:** Assessment schedule.

Visit schedules	
Visit 0 (screening)	Patient information (T)
	Study consent (T)
	Preassessment (T)
	Background information (T)
	Q-PLP (T)—includes PRI
	PDI (T)
	EQ5D-5L (T)
	PSEQ-2 (T)
	PCS-SF (T)
	PHQ-2 (T)
	EXPECT-SF (T)
Randomization	The therapist provides the monitor of the study with anonymized information about level of amputation, baseline PLP, investigation site of the enrolled patient; the monitor communicates back the assigned treatment
Visit 1	Treatment session (T)
	Q-PLP (E)—includes PRI
	OAT (E)
	EXPECT-SF (E)
	HCCQ-SF (E)
Visit 2-14	Treatment session (T)
	Q-PLP (E)—includes PRI
Visit 15	Treatment session (T)
	Q-PLP (E)—includes PRI
	PDI (E)
	EQ5D-5L (E)
	PSEQ-2 (E)
	PCS-SF (E)
	PHQ-2 (E)
	PGIC (E)
	HCCQ-SF (E)
Follow-up (1, 3, and 6 mo)	Q-PLP (E)—includes PRI
	PDI (E)
	EQ5D-5L (E)
	PSEQ-2 (E)
	PCS-SF (E)
	PHQ-2 (E)

The letter in brackets indicates whether the therapist (T) or the evaluator (E) is responsible of the item.

Abbreviations: EQ-5D-5L, EuroQol-5D-5L; EXPECT-SF, Expectations for Complementary and Alternative Medicine Treatments Short Form; HCCQ, Health Care Climate Questionnaire; OAT, Opinion About Treatment; PCS-SF, Pain Catastrophizing Scale Short Form; PDI, Pain Disability Index; PGIC, Patients Global Impression of Change; PHQ-2, 2-item Patient Health Questionnaire; PSEQ-2, 2-item Pain Self-Efficacy Questionnaire; Q-PLP, Questionnaire for Phantom Limb Pain. It includes the Short-Form McGill Pain Questionnaire, which is used to calculate the Pain Rating Index (PRI).

### 2.2 Randomization and masking

Participants were randomized in a 2:1 allocation ratio, with twice as many individuals receiving the PME treatment, according to the minimization scheme for optimal participant allocation.^31^ The minimization process, facilitated by an open-source desktop application, was operated by the trial monitor (E.L.) upon enrolment. At each research site, a therapist evaluated participant eligibility during the initial visit. Eligible participants were assigned a unique ID, indicating the research site. This ID, along with minimization factors, namely level of amputation and the numeric rating scale (NRS) value of the phantom limb pain, was shared with the study monitor, which generated the participant's group allocation based on these inputs. The treatment group assignment was relayed back to the administering therapist. The design was double-blinded, as participants were informed that the treatment received had been proven effective in previous studies,^21^ and were unaware of the existence of an active control arm. Evaluators did not take part in providing treatment and were blinded to which treatment was received by the interviewed participant.

### 2.3 Procedures

**Figure 1.**
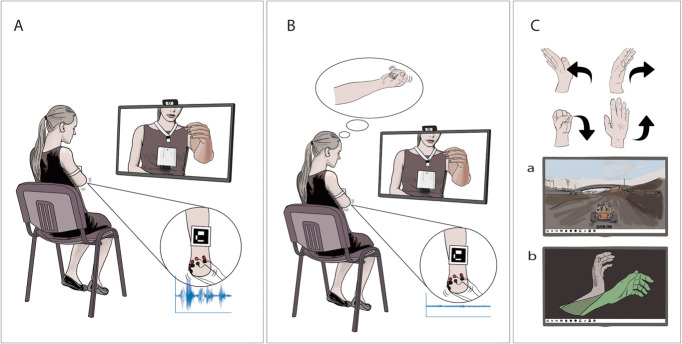
Interventions and treatment components. Representation of the clinical investigation device during the AR training in the PME group (panel A) and the PMI group (panel B). Myoelectric signals are acquired through surface electrodes by a differential amplifier. A webcam records the participant and the video is displayed on a computer monitor with a virtual arm or leg superimposed on a marker. In the magnification of the stump (visible in both panels A and B), it is possible to see how the PME treatment gives rise to detectable myoelectric signals, whereas PMI does not. Panel C is a representation of the other components of the training program. The participant trains with serious gaming (a) and with the TAC test which takes place in VR (b) by performing or imagining phantom motions (top part of panel C). Abbreviations: PME, Phantom Motor Execution; PMI, Phantom Motor Imagery; AR, Augmented Reality; TAC test, Targeted Achievement Control test.

All therapists were introduced to the technology with at least one practical demonstration by E.L. or M.O.C. These therapists then conducted the interventions independently at their outpatient clinics. E.L. monitored the correct execution of the study protocol. To improve adherence, the frequency of sessions was chosen by the participant and the therapists' availability, with options of 1, 2, or 5 times per week, yielding a total patient duration that ranged between 28 and 40 weeks. Both groups used the same equipment setup (Fig. 1), differing only in the type of interaction with the virtual environments (execution (PME) or imagination (PMI)). Each treatment session lasted 2 hours, including the blinded outcome assessment at the end of session (Table 2). The specific movements to train were chosen based on the level of amputation of the participants and on the current level of phantom motor dexterity as described in the published protocol.^18^

### 2.4 Experimental treatment—phantom motor execution (PME)

In the PME treatment, myoelectric signals were recorded from the residual limb and decoded in real-time to execute tasks in virtual reality/augmented reality (VR/AR) environments. Participants controlled the virtual environment using phantom movements and received real-time feedback on their performance (Fig. 1A).

### 2.5 Control treatment—phantom motor imagery (PMI)

In the PMI treatment, participants imagined performing movements while observing them autonomously executed in VR/AR environments. Myoelectric signals were recorded to monitor potential muscle contractions (rather than decoding motor volition as is done in PME). On-screen warnings notified participants when contractions were detected. Therapists and participants were informed about the difference between PMI and PME and instructed to avoid performing the latter (Fig. 1B).

### 2.6 Outcomes

The primary outcome was the change in PLP intensity measured by the difference in the Pain Rating Index (PRI) between baseline (visit 0) and end of treatment (visit 15). The PRI is a continuous variable computed as the sum of the scores for all descriptors of the Short Form of the McGill Pain Questionnaire (SF-MPQ).^24^ Each pain descriptor describes one of 15 different qualities of pain and related symptoms and is rated on an intensity scale as 0 = none, 1 = mild, 2 = moderate, or 3 = severe, giving a PRI that ranges from 0 to 45. Secondary outcomes considered different aspects related to PLP and consisted of the Patient Global Impression of Change (PGIC), which is a single-item survey to determine the patient's perception of treatment effectiveness and is rated on a 7-point scale from 'no change (or condition has worsened)' to 'a great deal better' (range 1-7); the PRI change between baseline and follow-up assessments; the PRI defined as a dichotomous variable, to indicate the presence of a clinically meaningful reduction in pain (CMRP; >50% pain decrease^29^); the Weighted Pain Distribution (WPD), which aims at quantifying the time-varying nature of chronic pain by summing scores of pain ratings during the day, weighted by the amount of time spent in each pain level (range 1-5) and PLP intensity on the NRS.

Additional exploratory outcomes included pain frequency, quality of pain, intrusion of pain in activities of daily living and sleep, disability associated with pain, pain self-efficacy, mood, presence of catastrophizing thinking, health-related quality of life, expectancy of benefit, quality of the patient–provider relationship, and judgment about the credibility of the used treatment. In addition, pain medication changes were recorded at each treatment session. Outcomes were evaluated by the blind evaluators in person at the end of every treatment session. Outcomes were also evaluated at 1, 3, and 6 months posttreatment. To incentivize the completion of the follow-up, the patients were given the choice to participate in these assessments at the clinic or using a phone interview. When possible, follow-up assessments were also conducted with participants who had discontinued the treatment. Lists of the assessments conducted at each visit are presented in Table 2. Details about the outcome measures are provided in the published study protocol^18^ and supplementary appendix.

### 2.7 Statistical analyses

The primary null hypothesis was that no significant between-group difference exists in the change in PRI score (derived from the SF-MPQ) between baseline and end of treatment. The sample size was determined based on a prior single-arm clinical trial of PME, reporting a 51% relative mean improvement in PRI.^25^ Given that this trial involved 2 groups and we anticipated some improvement in the control group, we deemed a 4-point difference between the groups (representing a reduction above 30%) to be both adequate and indicative of a CMRP. The 30% value was derived from a recalculation of the SF-MPQ results from the previous study adopting the 4-point validated scale; for more information, see the statistical analysis plan (SAP).^17^ To detect this difference with 80% power using a 2-sided Fisher nonparametric permutation test at a 5% significance level, we required a minimum of 66 participants (44 in the experimental group and 22 in the control group), including an allowance for potential withdrawals at a rate of 10% (6 participants). The sample size calculation was conducted using SAS 9.2 PROC.

For the main analyses, the 2 randomized groups were compared using Fisher nonparametric permutation test for continuous variables, Fisher exact test for dichotomous variables, the Mantel–Haenszel chi-square test for ordered categorical variables, and the Pearson chi-square test for nonordered categorical variables. All main analyses were performed on an intent-to-treat (ITT) basis, and data were imputed using stochastic regression modeling.

We performed predefined sensitivity analyses on the primary outcome to explore the effect of baseline characteristics on the estimated treatment effect and the impact of the stochastic regression imputation method by using Last Observation Carried Forward (LOCF) imputation and by analyzing the full analysis set. Complementary analyses were performed on the per-protocol (PP) population and prespecified subgroups.

In the confirmatory analyses, we used a fixed sequential method to control for type I error. Under this approach, if a test yields a significant result at the 5% significance level, the entire alpha level is passed onto the subsequent test in the sequence until a nonsignificant result is obtained. In short, we used a step-by-step approach to testing, carrying the same statistical 'threshold for significance' from one test to the next until a test failed to meet this threshold. All the significant tests preceding the first nonsignificant test are deemed confirmatory. We used this hierarchical testing procedure to examine both primary and secondary outcomes, whereas all other analyses were treated as exploratory. All analyses were performed with custom MATLAB scripts. MATLAB version 23.2.0 was used for all analyses. The study was preregistered with ClinicalTrials.gov, NCT03112928.

### Role of the funding source

All funding bodies had no influence on trial design, conduct, analysis, manuscript preparation, or decision to submit the manuscript for publication.

## 3 Results

**Figure 2.**
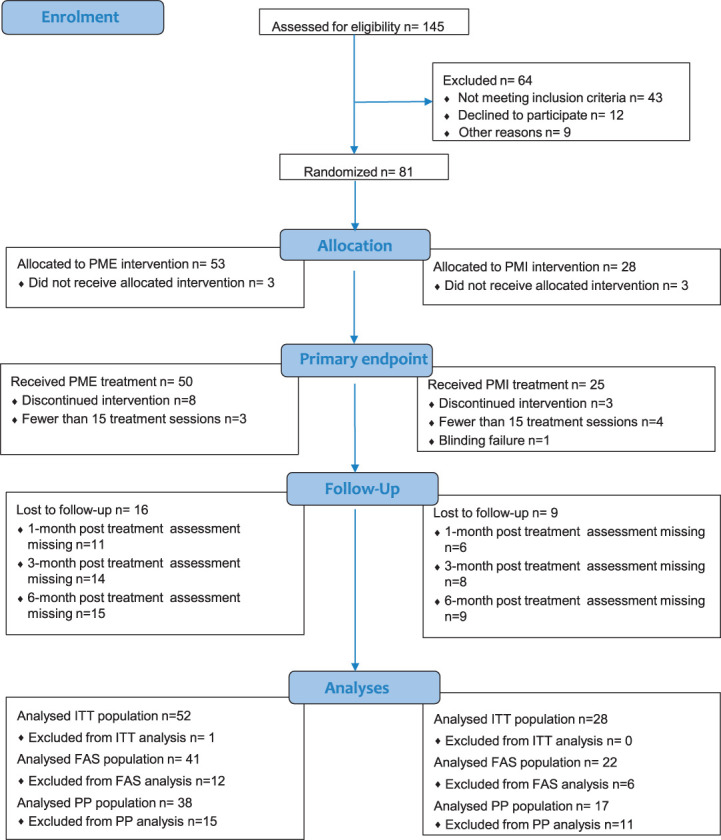
CONSORT flowchart. This flowchart contains the total number of participants per group that took part in the enrolment, randomisation, treatment received, and follow-up. The flowchart further details the subject disposition per treatment group observed in the analyses. Abbreviations: PME, Phantom Motor Execution; PMI, Phantom Motor Imagery; PP, per protocol; ITT, intent-to-treat; FAS, full analysis set.

Between May 8, 2017, and December 21, 2020, 145 patients were screened for eligibility (Table 1). Of the screened patients, 81 met the inclusion criteria and were randomized; data from one participant in the experimental group was irretrievably lost and omitted from the ITT population, resulting in 80 participants, of which 52 were allocated to the experimental group, and 28 to the control group. The trial flowchart is provided in Figure 2. The first treatment session of the trial took place on May 11, 2017, whereas the last session on March 12, 2021. The last follow-up assessment was carried out on September 20, 2021. The trial was ended upon reaching at least 60 participants (the pre-established sample size) that completed visit 15 and the final assessment.

Three participants in each group withdrew from the study before attending any treatment session. Of the remaining 74 participants, 55 completed the treatment without protocol deviations. Subject disposition is reported in Table S2, http://links.lww.com/PAIN/C120, which includes a breakdown of protocol deviations leading to exclusion from the ITT population. Baseline characteristics of the PP population are listed in Table S3, http://links.lww.com/PAIN/C120.

The participants' average age was 58.18 years, with similar heights and weights across both groups. The majority were male (72.5%), and the time since amputation and onset of phantom limb pain (PLP) varied, with mean values of 140.11 and 125.49 months (minimum: 4 months), respectively. Most participants (76.25%) had lower-limb amputation. The reasons for amputation included trauma (47.5%), cardiovascular/diabetes-related causes (31.25%), cancer (11.25%), and other or unknown reasons (10%). Participants used various types of prostheses, with 31.25% having none. The intensity of PLP was categorized as high (>4) for 56.25% of the subjects. The chosen treatment frequency was mostly twice a week (55%).

At baseline, the 2 groups exhibited some observable differences. For example, the percentage of male participants was higher in the PME group (78.85% vs 60.71%), and the time since amputation in the PMI group was on average longer (185.3 months vs 113.6 months). In addition, the onset of PLP in the PMI group was also longer (168.7 months vs 102.59 months). The distribution of reasons for amputation and types of prostheses also varied between the groups. The 2 groups were otherwise balanced (Table 3). History of previous treatments, previous medications, and concomitant medications are reported in Tables S6-S8, http://links.lww.com/PAIN/C120.

**Table 3:** Baseline characteristics.

Baseline characteristics (ITT population)			
*n (%) unless otherwise specified*	**PME (n=52)**	**PMI (n=28)**	**Overall (n=80)**
***Background information***			
Age at randomisation [y, mean (SD)]	58.21 (15.07)	58.11 (12.97)	58.18 (14.29)
Height [cm, mean (SD)]	173.4 (15.14)	170.25 (15.58)	172.3 (15.27)
Weight [kg, mean (SD)]	81.04 (15.94)	79.11 (17.66)	80.36 (16.48)
Sex [% male]	41 (78.85%)	17 (60.71%)	58 (72.5%)
Time since amputation [mo, mean (SD)]	113.6 (118.32)	185.3 (191.67)	140.11 (150.79)
Time since onset of PLP [mo, mean (SD)]	102.59 (117.3)	168.7 (189.03)	125.49 (148.37)
Reason for amputation			
Trauma	28 (53.85%)	10 (35.71%)	38 (47.5%)
Cardiovascular/diabetes	17 (32.69%)	8 (28.57%)	25 (31.25%)
Cancer	4 (7.69%)	5 (17.86%)	9 (11.25%)
Other	1 (1.92%)	5 (17.86%)	6 (7.5%)
Unknown	2 (3.85%)	0 (0%)	2 (2.5%)
Type of prosthesis			
None	15 (28.85%)	10 (35.71%)	25 (31.25%)
Active/myoelectric	12 (23.08%)	3 (10.71%)	15 (18.75%)
Cosmetic	2 (3.85%)	1 (3.57%)	3 (3.75%)
Passive	16 (30.77%)	12 (42.86%)	28 (35%)
Body-powered	7 (13.46%)	2 (7.14%)	9 (11.25%)
Time with current prosthesis (among users) [mo, mean (SD)]	28.94 (21.17)	35.28 (20.89)	28.94 (21.17)
Prosthesis usage (among users) [h, mean (SD)]	10.63 (4.89)	8.65 (5.4)	9.98 (5.1)
Minimization factors			
Level of amputation			
Transfemoral	26 (50%)	13 (46.43%)	39 (48.75%)
Transtibial	13 (25%)	8 (28.57%)	21 (26.25%)
Transmetatarsal	0 (0%)	1 (3.57%)	1 (1.25%)
Transhumeral	10 (19.23%)	5 (17.86%)	15 (18.75%)
Transradial	2 (3.85%)	1 (3.57%)	3 (3.75%)
Transmetacarpal	1 (1.92%)	0 (0%)	1 (1.25%)
Intensity of PLP			
high (>4)	30 (57.69%)	15 (53.57%)	45 (56.25%)
low (≤4)	20 (38.46%)	12 (42.86%)	32 (40%)
Investigational site			
1	4 (7.69%)	2 (7.14%)	6 (7.5%)
2	5 (9.62%)	4 (14.29%)	9 (11.25%)
3	5 (9.62%)	2 (7.14%)	7 (8.75%)
4	3 (5.77%)	1 (3.57%)	4 (5%)
5	3 (5.77%)	1 (3.57%)	4 (5%)
6	11 (21.15%)	5 (17.86%)	16 (20%)
7	14 (26.92%)	9 (32.14%)	23 (28.75%)
8	3 (5.77%)	1 (3.57%)	4 (5%)
9	4 (7.69%)	3 (10.71%)	7 (8.75%)
Chosen treatment frequency			
Once a week	10 (19.23%)	4 (14.29%)	14 (17.5%)
Twice a week	28 (53.85%)	16 (57.14%)	44 (55%)
5 times a week	14 (26.92%)	8 (28.57%)	22 (27.5%)
***Q-PLP***			
Intensity of PLP [NRS (0-10), mean (SD)]	4.48 (2.77)	4.04 (2.87)	4.38 (2.77)
Intensity of stump pain [NRS (0-10), mean (SD)]	1.44 (2.36)	1.25 (2.32)	1.38 (2.34)
Intensity of PLS [NRS (0-10), mean (SD)]	4.9 (3.67)	5.11 (3.55)	4.99 (3.63)
Intensity of voluntary phantom movements [NRS (0-10), mean (SD)]	5.62 (3.44)	6.75 (2.86)	6.03 (3.29)
Pain Rating Index (from the SF-MPQ) at baseline [PRI (0-45), mean (SD)]	14.5 (8.78)	14.71 (7.28)	14.7 (8.22)
Pain interference with sleep [NRS (0-10), mean (SD)]	4.12 (3.81)	6.61 (3)	4.96 (3.74)
Pain interference with daily-life activities [NRS (0-10), mean (SD)]	3.98 (2.95)	4.61 (3.19)	4.16 (3.03)
Pain interference with work [NRS (0-10), mean (SD)]	3.65 (3.08)	3.23 (3.33)	3.47 (3.16)
Presence of telescoping [NRS (0-10), mean (SD)]	4.61 (1.74)	4.33 (1.66)	4.51 (1.71)
Weighted Pain Distribution Index [WPD (0-5), mean (SD)]	1.73 (1.09)	1.92 (1.26)	1.81 (1.15)

The Q-PLP includes the short-form McGill Pain Questionnaire, which is used to calculate the Pain Rating Index (PRI). Abbreviations: PME, Phantom Motor Execution; PMI, Phantom Motor Imagery; PRI, Pain Rating Index; Q-PLP, Questionnaire for Phantom Limb Pain; NRS, Numeric Rating Scale; ITT, intent-to-treat, SF-MPQ = Short-Form McGill Pain Questionnaire.

### 3.1 Outcomes

Table 4 and Table 5 report estimated mean changes and treatment effects for the primary and secondary outcomes. Results were obtained by pooling statistical tests over 50 imputed datasets. Note that permutation tests do not provide an estimate for the difference of mean values; therefore, confidence intervals are not reported.^14^

**Table 4:** Primary, secondary, complementary, and sensitivity outcomes (ITT population).

	ITT population										
	PME				PMI				PME vs PMI		
	Mean change (SD_50_)	% Change	Mean SD (SD_50_)	Treatment effect (SD_50_)	Mean change (SD_50_)	% Change	Mean SD (SD_50_)	Treatment effect (SD_50_)	Diff. (mean ± SD_50_)	Treatment effect (SD_50_)	*P* (SD_50_)
***Primary outcome***											
**PRI**											
*Change (v0-v15)*	9.35 (0.22)	64.5%	10.67 (0.26)	0.88 (0.03)	10.03 (0.45)	68.2%	8.39 (0.31)	1.20 (0.07)	−0.68 (0.47)	−0.07 (0.05)	0.75 ± 0.12
***Secondary outcomes***											
**PGIC**											
*Visit 15*	4.16 (0.10)	N.A.	2.13 (0.06)	N.A.	3.94 (±0.18)	N.A.	1.93 (0.07)	N.A.	0.22 (0.22)	0.06 (0.04)	0.61 ± 0.23
**PRI**											
*Change (v0-1m)*	6.60 (0.45)	45.5%	13.08 (0.51)	0.51 (0.04)	7.86 (0.73)	53.4%	10.63 (0.51)	0.74 (0.09)	−1.25 (0.82)	−0.10 (0.07)	0.66 ± 0.18
*Change (v0-3m)*	5.90 (0.51)	40.7%	12.70 (0.53)	0.47 (0.05)	6.16 (0.69)	41.8%	11.81 (0.67)	0.52 (0.08)	−0.26 (0.81)	−0.02 (0.07)	0.82 ± 0.13
*Change (v0-6m)*	6.05 (0.28)	41.7%	11.87 (0.33)	0.51 (0.03)	6.68 (0.58)	45.4%	10.98 (0.55)	0.61 (0.07)	−0.63 (0.61)	−0.05 (0.05)	0.78 ± 0.12
**PLP WPD**											
*Change (v0-v15)*	0.40 (0.04)	23.2%	1.24 (0.05)	0.33 (0.04)	0.74 (0.07)	38.6%	1.31 (0.08)	0.57 (0.08)	−0.34 (0.09)	−0.27 (0.07)	0.27 ± 0.13
*Change (v0-1m)*	0.19 (0.04)	11.1%	1.46 (0.07)	0.13 (0.03)	0.66 (0.05)	34.2%	1.19 (0.06)	0.55 (0.06)	−0.47 (0.06)	−0.34 (0.04)	0.16 ± 0.05
*Change (v0-3m)*	0.20 (0.04)	11.7%	1.29 (0.05)	0.16 (0.04)	0.41 (0.08)	21.3%	1.10 (0.06)	0.37 (0.09)	−0.21 (0.10)	−0.17 (0.08)	0.49 ± 0.19
*Change (v0-6m)*	0.10 (0.05)	5.9%	1.28 (0.06)	0.08 (0.04)	0.07 (0.09)	3.7%	1.18 (0.08)	0.06 (0.08)	0.03 (0.12)	0.03 (0.09)	0.76 ± 0.18
**PLP NRS**											
*Change (v0-v15)*	1.97 (0.11)	44.0%	3.17 (0.10)	0.62 (0.04)	1.25 (0.18)	31.1%	3.48 (0.23)	0.36 (0.07)	0.72 (0.21)	0.22 (0.06)	0.37 ± 0.13
*Change (v0-1m)*	0.81 (0.16)	18.0%	3.34 (0.12)	0.24 (0.05)	0.19 (0.20)	4.6%	3.76 (0.21)	0.05 (0.05)	0.62 (0.24)	0.18 (0.07)	0.47 ± 0.18
*Change (v0-3m)*	0.98 (0.12)	21.8%	3.36 (0.10)	0.29 (0.04)	0.12 (0.25)	2.9%	4.03 (0.25)	0.03 (0.06)	0.86 (0.28)	0.24 (0.08)	0.33 (0.16)
*Change (v0-6m)*	0.44 (0.15)	9.8%	3.50 (0.18)	0.13 (0.05)	−0.13 (0.25)	−3.1%	3.86 (0.27)	−0.03 (0.06)	0.57 (0.26)	0.16 (0.07)	0.52 ± 0.19
***Sensitivity analyses***											
**LOCF PRI**											
*Change (v0-v15)*	8.04 (10.37)	0.554		0.77	6.39 (9.65)	0.434	0.66		1.65		0.49
**FAS PRI**											
*Change (v0-v15)*	9.17 (9.42)	0.649		0.97	9.23 (8.58)	0.663	1.08		−0.06		0.97
**ANCOVA PRI**											
*Change (v0-v15)*									−0.32 (0.46)	—	

The table contains the results for the main analysis of the primary outcome measure, ie, the PRI derived from the Short-Form McGill Pain Questionnaire, which assesses qualitative aspects of PLP. In addition, the table also includes the main analysis of secondary outcomes. These tests were performed with the Fisher permutation on the ITT population. Fifty datasets were imputed and analyzed for every outcome variable.

Sensitivity analyses were conducted to assess the impact of the imputation method by analyzing the ITT population using the LOCF approach and by analyzing the FAS population. These analyses were also carried out using the Fisher permutation test. ITT population was also used to build 50 linear models with the formula ∆PRI ∼ Treatment + ampLocation + NRS PLPv0 + PRIv0, which were tested using ANCOVA to carry out an adjusted analysis. Treatment effects in this case were calculated with the partial eta squared (as opposed to the Cohens *d* adopted in all the other cases). Histograms showing the distribution of PRI between-group differences and *P*-values are shown in Figure S14, http://links.lww.com/PAIN/C120.

Abbreviations: ITT, intent-to-treat; PME, Phantom Motor Execution; PMI, Phantom Motor Imagery; PRI, Pain Rating Index; Diff., difference between PME and PMI; LOCF, Last Observation Carried Forward; FAS, full analysis set; NRS, Numeric Rating Scale; WPD, Weighted Pain Distribution; Q-PLP, Questionnaire about Phantom Limb Pain; PGIC, Patient Global Impression of Change; v0, visit 0 (baseline); v15, visit 15 (end of treatment); 1m, 1 mo follow-up; 3m, 3 mo follow-up; 6m, 6 mo follow-up; ampLocation, amputation location (upper/lower limb); SD, SD inherent to the data; SD_50_= SD across the 50 imputed datasets, which highlights the imputation-induced variability.

**Table 5:** Primary, secondary, complementary, and sensitivity outcomes (PP population).

PP population								
	Change (mean ± SD)	% Change	Treatment effect	Change (mean ± SD)	% Change	Treatment effect	Diff	Treatment effect
**PRI**								
*Change (v0-v15)*	8.89 (9.55)	62.9%	0.93	10.12 (9.49)	67.7%	1.07	−1.22	−0.13
**PGIC**								
*Visit 15*	4.17 (2.19)	N.A.	N.A.	3.95 (2.01)	N.A.	N.A.	0.22	0.02
**PRI**								
*Change (v0-1m)*	7.11 (11.61)	47.9%	0.61	7.94 (12.15)	53.1%	0.65	−0.83	−0.07
*Change (v0-3m)*	6.33 (10.04)	42.4%	0.63	8.00 (12.97)	51.3%	0.62	−1.67	−0.15
*Change (v0-6m)*	5.25 (11.10)	37.1%	0.47	7.13 (13.05)	45.7%	0.55	−1.88	−0.16
**PLP WPD**								
*Change (v0-v15)*	0.50 (1.12)	26.9%	0.45	0.87 (1.35)	44.9%	0.64	−0.37	−0.31
*Change (v0-1m)*	0.25 (1.35)	13.5%	0.19	0.82 (1.28)	42.2%	0.64	−0.57	−0.43
*Change (v0-3m)*	0.27 (1.22)	14.0%	0.22	0.54 (1.18)	28.1%	0.46	−0.27	−0.23
*Change (v0-6m)*	0.24 (1.14)	12.5%	0.21	0.08 (1.22)	4.3%	0.07	0.15	0.13
**PLP NRS**								
*Change (v0-v15)*	1.89 (2.98)	40.7%	0.63	1.82 (3.30)	39.7%	0.55	0.07	0.02
*Change (v0-1m)*	1.00 (2.70)	20.9%	0.37	1.00 (3.48)	21.8%	0.29	0	0
*Change (v0-3m)*	1.61 (2.75)	31.9%	0.58	0.93 (3.69)	20.9%	0.25	0.67	0.22
*Change (v0-6m)*	0.69 (3.11)	14.5%	0.22	0.13 (4.07)	3.0%	0.03	0.55	0.16

Complementary analyses carried out for the primary and all secondary outcome variables for the PP population. The main outcome variable is the PRI, derived from the Short-form McGill Pain Questionnaire. Abbreviations: PP, per protocol; PME, Phantom Motor Execution; PMI, Phantom Motor Imagery; PRI, Pain Rating Index; Diff., difference between PME and PMI; LOCF, Last Observation Carried Forward; FAS, full analysis set; NRS, Numeric Rating Scale; WPD, Weighted Pain Distribution; Q-PLP, Questionnaire about Phantom Limb Pain; PGIC, Patient Global Impression of Change; v0, visit 0 (baseline); v15, visit 15 (end of treatment); 1m, 1-mo follow-up; 3m, 3-mo follow-up; 6m, 6-mo follow-up; ampLocation, amputation location (upper/lower limb); SD, standard deviation inherent to the data.

No superiority of PME over PMI was found. The main unadjusted analysis of the primary outcome showed a mean between-group difference of 0.68 ± 0.47 (Cohen *d* 0.07 ± 0.05; *P* = 0.75 ± 0.12). As no significant improvement of PME over PMI was found for the primary outcome variable, all ensuing comparisons are considered exploratory; therefore, *P*-values are not reported.

**Figure 3.**
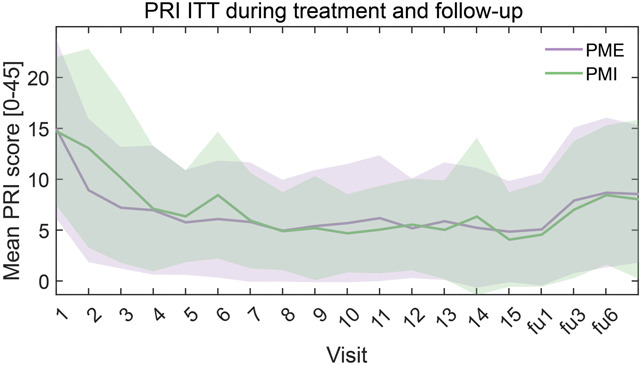
PRI trend (ITT) The trend of mean (lines) and SD (shaded areas) of PRI for PME (lilac) and PMI (green). The trends of pain scores are reported throughout the study and follow-up sessions. Visit 0: baseline; visits 1-15: treatment sessions; +1 m, +3 m and +6 m: 1-, 3-, and 6-month follow-up assessments. Total range of PRI score is 0 to 45. The treatment effect was assessed as the difference in pain score between baseline and endpoints. No difference in treatment efficacy was found between groups. Abbreviations: ITT, intent-to-treat; PME, Phantom Motor Execution; PMI, Phantom Motor Imagery; PRI, Pain Rating Index.

We found effects for the primary outcome from baseline to last treatment session with a reduction in PRI (derived from the SF-MPQ) in PME 64.5% and in PMI 68.2% (Table 4). The secondary outcomes showed similar improvements as the primary outcome, i.e., positive effects of both interventions with no superiority of PME, across different measures. Pain Rating Index (PRI), over 1, 3, and 6 months posttreatment, showed a reduction with percentage changes ranging from 18.0% to 45.5% for PME and from 4.6% to 53.4% for PMI. In the PLP Weighted Pain Distribution (WPD) and PLP Numeric Rating Scale (NRS), both groups exhibited reductions at various time points, with percentage changes ranging from 5.9% to 38.6% for PME and from 3.7% to 44.9% for PMI. The trend of PRI scores over time for the ITT population is presented in Figure 3 (for comparison, the trend for the PP population is shown in Fig. S6, http://links.lww.com/PAIN/C120). Generally, we observed an increase in pain levels from the end of treatment to the 1-month follow-up, signifying a partial rebound. Subsequently, the pain levels remained consistent, and by the 6-month follow-up, they settled at a point lower than the baseline for both treatment modalities. The complementary analyses in the PP population also reflected similar trends, with no significant differences between groups.

**Figure 4.**
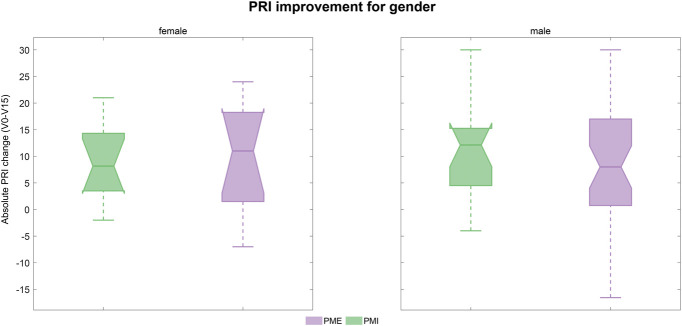
PRI improvement according to sex (ITT population): Boxplots of the improvements in PRI score (difference between baseline and visit 15) in the 2 treatment groups according to sex. Results refer to the ITT population and are shown in green for PMI and lilac for PME. Abbreviations: PRI, Pain Rating Index; ITT, intent-to-treat; PME, Phantom Motor Execution; PMI, Phantom Motor Imagery.

**Figure 5.**
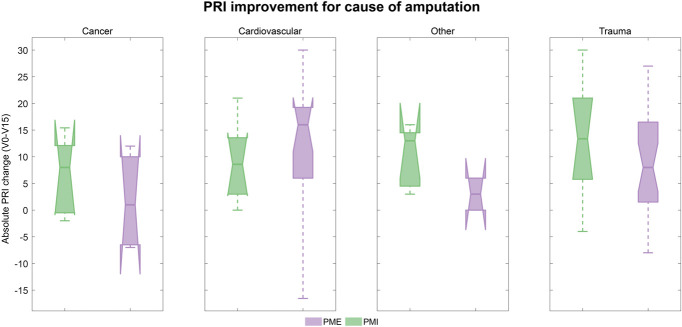
PRI improvement according to cause of amputation (ITT population): Boxplots of the improvements in PRI score (difference between baseline and visit 15) in the 2 treatment groups for the ITT population according to amputation cause. Results refer to the ITT population and are shown in green for PMI and lilac for PME. Abbreviations: PRI, Pain Rating Index; ITT, intent-to-treat; PME, Phantom Motor Execution; PMI, Phantom Motor Imagery.

**Figure 6.**
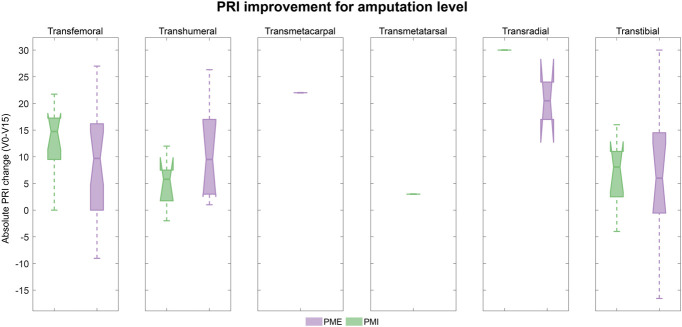
PRI improvement according to amputation level (ITT population): Boxplots of the improvements in PRI score (difference between baseline and visit 15) in the 2 treatment groups according to amputation level. Results refer to the ITT population and are shown in green for PMI and lilac for PME. Participants with transradial and transmetacarpal amputations in both treatment groups seem to achieve a better improvement on phantom pain. However, the number of subjects was limited in these groups (n = 1 transmetacarpal and n = 4 transradial). Abbreviations: PRI, Pain Rating Index; ITT, intent-to-treat; PME, Phantom Motor Execution; PMI, Phantom Motor Imagery.

**Figure 7.**
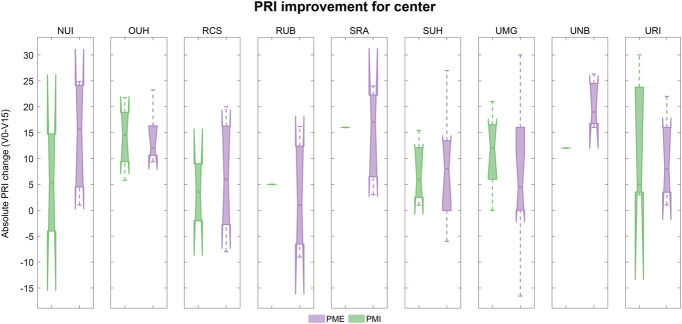
PRI improvement according to investigational site (ITT population): Boxplots of the improvements in PRI score (difference between baseline and visit 15) in the 2 treatment groups according to investigational site. Results refer to the ITT population and are shown in green for PMI and lilac for PME. Abbreviations: PRI, Pain Rating Index; ITT, intent-to-treat; PME, Phantom Motor Execution; PMI, Phantom Motor Imagery.

Predefined subgroup analyses assessed the differences in the effect of the treatments depending on sex, amputation level, cause of amputation, and treatment clinic. Differences between subgroups were negligible, and results are shown for the ITT in Figures 4-7. Medians, interquartile ranges, and min and max of PRI improvements for each subgroup are reported in Table 6.

**Table 6:** PRI improvements in subgroups for ITT population.

PRI score in subgroups for ITT population										
	PME					PMI				
	**Median**	**IQR**	**Min**	**Max**	**n**	**Median**	**IQR**	**Min**	**Max**	**n**
**Sex**										
female	11.0	16.8	−7.0	24.0	11.0	8.2	10.8	−2.0	21.0	11.0
male	8.0	16.3	−16.6	30.0	41.0	12.1	10.8	−4.0	30.0	17.0
**Amputation cause**										
Cancer	1.0	16.5	−7.0	12.0	4.0	8.0	12.6	−2.0	15.4	5.0
Cardiovascular/diabet.	16.0	13.3	−16.6	30.0	17.0	8.6	10.6	0.0	21.0	8.0
Trauma	8.0	15.0	−8.0	27.0	27.0	13.4	15.2	−4.0	30.0	10.0
Other	3.0	6.0	0.0	6.0	2.0	13.0	10.0	3.0	16.0	5.0
**Investigation site**										
Site 1	15.7	19.6	1.0	24.9	4.0	5.4	18.7	−4.0	14.7	2.0
Site 2	12.0	5.7	9.4	23.2	5.0	14.5	9.5	5.8	21.7	4.0
Site 3	6.0	19.0	−8.0	20.0	5.0	3.5	11.0	−2.0	9.0	2.0
Site 4	1.0	18.9	−9.0	16.2	3.0	5.0	0.0	5.0	5.0	1.0
Site 5	17.0	15.8	3.0	24.0	3.0	16.0	0.0	16.0	16.0	1.0
Site 6	8.0	13.5	−6.0	27.0	11.0	6.0	9.6	1.0	15.4	5.0
Site 7	4.5	16.0	−16.6	30.0	14.0	12.1	10.5	0.0	21.0	9.0
Site 8	19.0	7.7	16.0	26.3	3.0	12.0	0.0	12.0	12.0	1.0
Site 9	8.0	12.5	1.0	22.0	4.0	5.0	20.3	3.0	30.0	3.0
**Amputation level**										
Transmetacarpal	22.0	0.0	22.0	22.0	1.0	n.a.				
Transmetatarsal	n.a.					3.0	0.0	3.0	3.0	1.0
Transradial	20.5	7.0	17.0	24.0	2.0	30.0	0.0	30.0	30.0	1.0
Transhumeral	9.5	14.0	1.0	26.3	10.0	5.8	5.8	−2.0	12.0	5.0
Transtibial	6.0	15.0	−16.6	30.0	13.0	8.1	8.5	−4.0	16.0	8.0
Transfemoral	9.7	16.2	−9.0	27.0	26.0	14.7	7.8	0.0	21.7	13.0

PRI improvements between baseline and visit 15 in different subgroups of the ITT population, divided by treatment group. Abbreviations: PRI, Pain Rating Index (derived from the Short-form McGill Pain Questionnaire); ITT, intent-to-treat; PME, Phantom Motor Execution; PMI, Phantom Motor Imagery; IQR, interquartile range.

Thirty-seven participants in the PME group (71%, n = 52) and 19 in the PMI group (68%, n = 28) experienced CMRP for PRI (>50% reduction) with an odds ratio of 1.14 ± 0.19. The same analysis on the PP population showed that 27 participants in the PME group (71%, n = 38) and 11 in the PMI group (65%, n = 17) experienced a CMRP for PRI (odds ratio 1.34). Overall, the mean PRI reduction in the ITT population was 9.35 ± 0.22 for the PME group, with a large within-group effect size (Cohen *d* 0.88 ± 0.03), and 10.03 ± 0.45 for the PMI group, with an even larger within-group effect size (Cohen *d* 1.20 ± 0.07).

An examination of pain medication usage revealed that the majority of participants had been using pain medications (for at least 3 months) before the outset of the study. Interestingly, the group-level analysis of this usage did not indicate significant changes throughout the duration of the study, as shown in Tables S5-S9, http://links.lww.com/PAIN/C120.

Both groups achieved beneficial small to medium treatment effects across all exploratory outcome variables (see Table S10, http://links.lww.com/PAIN/C120). Most notably, pain catastrophizing, mood and interest/pleasure in routine activities, disability associated with pain, pain interference with sleep, work, and daily living appeared to be the outcomes where participants benefited most. A potential effect of PMI over PME was found for interference with sleep (Cohen *d* 0.52 ± 0.06), indicating a medium effect size favoring PMI. Similarly, the trend over time for nonpainful phantom sensations (Fig. S11, http://links.lww.com/PAIN/C120) showed that after starting from similar baselines, the PMI group consistently experienced more intense nonpainful phantom sensations compared with PME (Cohen *d* 0.54 ± 0.08). A critical aspect of the study's design was the successful masking of treatment expectations, evaluated with the Expectations for Complementary and Alternative Medicine Treatments (EXPECT) questionnaire, and credibility, evaluated with the Opinion About Treatment (OAT) questionnaire. Our analysis confirmed that there were no differences between the groups in these areas, reinforcing the integrity of the blinding process (Table S10, http://links.lww.com/PAIN/C120).

Both treatment groups demonstrated improvements in phantom limb pain frequency and telescoping, with no discernible superiority of one treatment over the other, as detailed in Tables S11, http://links.lww.com/PAIN/C120.

Table 7 is a summary of the absolute number and percentage of participants reporting specific McGill pain descriptors over baseline, end of treatment, and follow-up assessments. The data provide insight into the prevalence of pain types among participants rather than their severity. Overall, this table allows us to observe the descriptors that ceased to be reported or persisted over time. In addition, Table 8 presents the percentage changes in the incidence of these pain descriptors across the same time points, offering a clearer view of the shifts in reported sensations.

**Table 7:** McGill pain descriptors throughout the study for the FAS population.

	Visit 0			Visit 15			1M			3M			6M		
	Overall	PME	PMI	Overall	PME	PMI	Overall	PME	PMI	Overall	PME	PMI	Overall	PME	PMI
Throbbing	36	23	13	14	10	4	24	16	8	22	16	6	22	17	5
	45%	44.2%	46.4%	22.2%	24.4%	18.2%	37.5%	38.1%	36.4%	37.3%	41%	30%	39.3%	45.9%	26.3%
Shooting	38	23	15	9	6	3	14	11	3	14	10	4	18	13	5
	47.5%	44.2%	53.6%	14.3%	14.6%	13.6%	21.9%	26.2%	13.6%	23.7%	25.6%	20%	32.1%	35.1%	26.3%
Stabbing	48	31	17	23	15	8	24	17	7	26	18	8	27	20	7
	60%	59.6%	60.7%	36.5%	36.6%	36.4%	37.5%	40.5%	31.8%	44.1%	46.2%	40%	48.2%	54.1%	36.8%
Cramping	50	33	17	23	15	8	21	14	7	28	18	10	22	15	7
	62.5%	63.5%	60.7%	36.5%	36.6%	36.4%	32.8%	33.3%	31.8%	47.5%	46.2%	50%	39.3%	40.5%	36.8%
Sharp	40	26	14	15	10	5	19	15	4	22	16	6	14	8	6
	50%	50%	50%	23.8%	24.4%	22.7%	29.7%	35.7%	18.2%	37.3%	41%	30%	25%	21.6%	31.6%
Gnawing	29	22	7	9	8	1	17	12	5	17	14	3	16	11	5
	36.3%	42.3%	25%	14.3%	19.5%	4.5%	26.6%	28.6%	22.7%	28.8%	35.9%	15%	28.6%	29.7%	26.3%
Hot-burning	38	25	13	21	12	9	23	13	10	25	14	11	17	14	3
	47.5%	48.1%	46.4%	33.3%	29.3%	40.9%	35.9%	31%	45.5%	42.4%	35.9%	55%	30.4%	37.8%	15.8%
Aching	56	37	19	22	16	6	31	21	10	28	21	7	31	21	10
	70%	71.2%	67.9%	34.9%	39%	27.3%	48.4%	50%	45.5%	47.5%	53.8%	35%	55.4%	56.8%	52.6%
Heavy	42	25	17	15	10	5	17	13	4	21	13	8	17	11	6
	52.5%	48.1%	60.7%	23.8%	24.4%	22.7%	26.6%	31%	18.2%	35.6%	33.3%	40%	30.4%	29.7%	31.6%
Tender	29	19	10	6	2	4	15	9	6	17	11	6	22	16	6
	36.3%	36.5%	35.7%	9.5%	4.9%	18.2%	23.4%	21.4%	27.3%	28.8%	28.2%	30%	39.3%	43.2%	31.6%
Splitting	30	18	12	8	6	2	8	5	3	12	7	5	10	6	4
	37.5%	34.6%	42.9%	12.7%	14.6%	9.1%	12.5%	11.9%	13.6%	20.3%	17.9%	25%	17.9%	16.2%	21.1%
Tiring-exhausting	57	35	22	18	13	5	27	19	8	25	17	8	25	18	7
	71.3%	67.3%	78.6%	28.6%	31.7%	22.7%	42.2%	45.2%	36.4%	42.4%	43.6%	40%	44.6%	48.6%	36.8%
Sickening	9	4	5	6	4	2	5	4	1	2	1	1	2	2	0
	11.3%	7.7%	17.9%	9.5%	9.8%	9.1%	7.8%	9.5%	4.5%	3.4%	2.6%	5%	3.6%	5.4%	0%
Fearful	22	17	5	6	4	2	11	9	2	8	6	2	7	6	1
	27.5%	32.7%	17.9%	9.5%	9.8%	9.1%	17.2%	21.4%	9.1%	13.6%	15.4%	10%	12.5%	16.2%	5.3%
Punishing-cruel	27	19	8	4	2	2	8	6	2	7	4	3	7	3	4
	33.8%	36.5%	28.6%	6.3%	4.9%	9.1%	12.5%	14.3%	9.1%	11.9%	10.3%	15%	12.5%	8.1%	21.1%

This table presents the absolute numbers and percentages of participants reporting each McGill pain descriptor at baseline (visit 0), end of treatment (visit 15), and follow-up assessments at 1 mo (1M), 3 mo (3M), and 6 mo (6M). The data illustrate the prevalence of various pain types over time, providing insight into descriptors that were either resolved or persisted. Results are displayed overall and by treatment group. The FAS population includes all subjects in the intent-to-treat (ITT) population without imputation, ensuring percentages reflect the prevalence among those still actively participating in the study.

Abbreviations: ITT, intent-to-treat; FAS, full analysis set; PME, Phantom Motor Execution; PMI, Phantom Motor Imagery.

**Table 8:** Percentage changes in McGill pain descriptors from baseline across all visits for the FAS population.

	V15-V0			FU1-V0			FU3-V0			FU6-V0		
	Overall	PME	PMI	Overall	PME	PMI	Overall	PME	PMI	Overall	PME	PMI
Throbbing	−22.80%	−19.80%	−28.20%	−7.50%	−6.10%	−10%	−7.70%	−3.20%	−16.40%	−5.70%	1.70%	−20.10%
Shooting	−33.20%	−29.60%	−40%	−25.60%	−18%	−40%	−23.80%	−18.60%	−33.60%	−15.40%	−9.10%	−27.30%
Stabbing	−23.50%	−23%	−24.30%	−22.50%	−19.10%	−28.90%	−15.90%	−13.40%	−20.70%	−11.80%	−5.50%	−23.90%
Cramping	−26%	−26.90%	−24.30%	−29.70%	−30.20%	−28.90%	−15%	−17.30%	−10.70%	−23.20%	−23%	−23.90%
Sharp	−26.20%	−25.60%	−27.30%	−20.30%	−14.30%	−31.80%	−12.70%	−9%	−20%	−25%	−28.40%	−18.40%
Gnawing	−22%	−22.80%	−20.50%	−9.70%	−13.70%	−2.30%	−7.50%	−6.40%	−10%	−7.70%	−12.60%	1.30%
Hot-burning	−14.20%	−18.80%	−5.50%	−11.60%	−17.10%	−0.90%	−5.10%	−12.20%	8.60%	−17.10%	−10.30%	−30.60%
Aching	−35.10%	−32.20%	−40.60%	−21.60%	−21.20%	−22.40%	−22.50%	−17.40%	−32.90%	−14.60%	−14.40%	−15.30%
Heavy	−28.70%	−23.70%	−38%	−25.90%	−17.10%	−42.50%	−16.90%	−14.80%	−20.70%	−22.10%	−18.40%	−29.10%
Tender	−26.80%	−31.60%	−17.50%	−12.90%	−15.10%	−8.40%	−7.50%	−8.30%	−5.70%	3%	6.70%	−4.10%
Splitting	−24.80%	−20%	−33.80%	−25%	−22.70%	−29.30%	−17.20%	−16.70%	−17.90%	−19.60%	−18.40%	−21.80%
Tiring-exhausting	−42.70%	−35.60%	−55.90%	−29.10%	−22.10%	−42.20%	−28.90%	−23.70%	−38.60%	−26.70%	−18.70%	−41.80%
Sickening	−1.80%	2.10%	−8.80%	−3.50%	1.80%	−13.40%	−7.90%	−5.10%	−12.90%	−7.70%	−2.30%	−17.90%
Fearful	−18%	−22.90%	−8.80%	−10.30%	−11.30%	−8.80%	−13.90%	−17.30%	−7.90%	−15%	−16.50%	−12.60%
Punishing-cruel	−27.50%	−31.60%	−19.50%	−21.30%	−22.20%	−19.50%	−21.90%	−26.20%	−13.60%	−21.30%	−28.40%	−7.50%

This table presents the percentage changes in the incidence of each McGill pain descriptor from baseline (V0) to the end of treatment (V15) and follow-up assessments at 1 mo (FU1), 3 mo (FU3), and 6 mo (FU6). The data illustrate shifts in reported sensations over time, providing insight into descriptors that either resolved or persisted. Results are displayed overall and by treatment group. The FAS population includes all subjects in the intent-to-treat (ITT) population without imputation, ensuring percentages reflect the prevalence among those still actively participating in the study.

Abbreviations: ITT, intent-to-treat; FAS, full analysis set; PME, Phantom Motor Execution; PMI, Phantom Motor Imagery.

At baseline, the most common descriptors of pain reported by participants in the study were “stabbing,” “cramping,” “aching,” and “tiring-exhausting,” with over 60% of participants across groups identifying these sensations. Notably, “aching” and “tiring-exhausting” were the most prevalent, indicating a high level of discomfort and fatigue associated with their condition. The distribution was consistent across both treatment groups, PME and PMI, suggesting similar baseline pain experiences.

At the end of treatment, the most notable decreases were seen in “tiring-exhausting” (−42.7% overall), “aching” (−35.1% overall), and “shooting” (−33.2% overall), suggesting a substantial number of participants no longer reported these sensations. No substantial difference was observed between the groups at the end of the treatment, with 9 of the 15 descriptors found slightly less often in the PMI group. Descriptors such as “throbbing”, “shooting”, and “tiring-exhausting” were found less often in PMI, and “hot-burning”, “fearful”, and “punishing-cruel” found less often in PME.

At the 1-month follow-up, improvements persisted, although to a lesser extent, with “shooting” pain remaining significantly reduced overall at −25.6%. The proportion of descriptors prevalence remained the same (9 of 15 lower for PMI). By the 3-month (FU3) and 6-month follow-ups (FU6), the overall reductions in descriptors remained notable but diminished, with the PMI group maintaining larger reductions in descriptors such as “shooting” and “tiring-exhausting”, and PME for “fearful” and “punishing-cruel”.

No adverse effects were reported.

## 4 Discussion

The current study, designed to test for the superiority of overt motor execution (PME) over imagined phantom movements (PMI), using XR, found no superiority of PME over PMI in treating PLP. The study conducted was one of the largest international, multicenter, double-blinded RCTs for nonpharmacological treatments of chronic PLP to date. Secondary to this, similar levels of improvement in the primary outcome were experienced in both PME and PMI, with an overall mean PLP reduction of 65%-68% (PRI).

This improvement was greater than previously reported in a single group study investigating the efficacy of PME on PLP after upper limb amputations.^25^The results from this study suggest that both PME and PMI are likely to offer meaningful PLP relief to most patients; however, further investigation is needed to determine the clinical meaningfulness of these improvements. As previously seen, we observed a trend of initial partial pain rebound after treatment cessation^27^ which eventually stabilized to levels lower than baseline over an extended follow-up period. Similarly to the primary outcome, the secondary outcomes, also improved for both interventions with no superiority of PME. For example, pain catastrophizing, mood, and interest/pleasure in routine activities, disability associated with pain, pain interference with sleep, work, and activities of daily living all improved (Tables 4 and 5). Importantly, none of the participants reported adverse effects. The latter compares favorably to the outcomes for pharmacological treatments. A Cochrane review^1^ concluded that there was little evidence of benefit from most substances examined, with the exception of morphine, where around 40% of patients achieved >50% pain reduction but secondary outcomes did not improve, and many adverse effects were reported.

Numerous studies have used XR-supported treatments to alleviate chronic pain. A 2022 systematic review and meta-analysis^10^ found that interventions using XR had a significant effect on reducing pain intensity, with an average effect size of 1.60 (95% CI 0.83-2.36; *P* < 0.001) across all the studies.^34^ Our results are in line with this meta-analysis as we found no difference between groups but reported some of the largest and longest-lasting reductions of PLP in an RCT. This suggests that the way in which XR is employed is important and should be further investigated.

One possible interpretation of our findings is that both PME and PMI engage similar pain modulatory mechanism(s). Motor imagery, like motor execution, has been shown to increase corticospinal excitability, induce plasticity in the spinal cord, and enhance skeletal muscle excitability.^11,12^ Despite the suppression of overt motor execution in PMI, the significant overlap in cortical activation during motor intention and planning for both executing and imagining movements suggests that these treatments may engage similar pain modulatory mechanisms.

Another way to interpret the present results could be that both treatments provide pain relief of similar magnitude despite engaging distinct modulatory mechanisms. The PME group was required to produce distinct muscular contractions, classified by machine learning algorithms, which may have provoked a slow learning process for specific motor output patterns. Conversely, the PMI group trained without such requirements and did not receive performance feedback. Moreover, it is suggested that at least part of the subcortical activation in PMI is of an inhibitory nature to suppress overt motor execution.

Given the lack of observed differences in efficacy between the 2 therapies and lack of a passive placebo group, it is challenging to determine which interpretation of our findings holds more validity. This ambiguity underscores the complexity of understanding and managing PLP and highlights a significant gap in the current knowledge surrounding the etiology.

Although our study presents encouraging results, we recognize several limitations that warrant careful consideration. Firstly, despite using an active control, we cannot entirely exclude the influence of placebo effects. However, the substantial PLP reduction (>60%) observed in our study far exceeds the typical 20% improvement seen in placebo groups of pharmacological therapies for PLP. This improvement aligns more closely with improvements often reported in the experimental arms of pharmacological RCTs.^23^ Such a marked difference, in conjunction with the enduring treatment effects beyond the intervention, suggests that placebo effects likely played a minor role in our findings. Similarly, distraction, a common pain management strategy, is unlikely to solely account for the persistent treatment effects observed up to 6 months posttreatment.

Our study's design, allowing participants to choose their treatment frequency, deviates from the conventional method of assessing outcomes at fixed times postrandomization. This was done with the main goal to enhance adherence, minimize dropouts and accommodate diverse scheduling needs across different investigation sites. As we expected, our subgroup analysis revealed no significant differences in outcomes based on treatment frequency (Fig. S16, http://links.lww.com/PAIN/C120).

Despite these design decisions aimed at minimizing attrition, we still encountered a larger-than-anticipated dropout rate, exceeding the 10% estimated during our sample size calculations. This underscores the importance of developing enhanced strategies to retain patients in future studies. Nonetheless, we addressed the issue created by missing data by using stochastic regression imputation, ensuring that the results remained unbiased in this regard. Another possible limitation of our study could be the influence of contextual factors. For example, therapists and patients spent an average of 30 hours together, creating a strong therapeutic alliance.^29^ We evaluated the patient–provider relationship using a specific survey (the HCCQ), and found high scores with no difference between groups. Such strength in the therapeutic alliance could contribute to demand characteristics—the subtle cues that convey expected outcomes within experimental settings.^25^ As a result, the observed effects may not be solely attributable to the intervention but could also be shaped by the expectations of both therapists and patients. In this study, we attempted to mitigate this confounder by using blind evaluators. Furthermore, studies where self-administered XR treatments took place at home have reported improvements in pain of comparable magnitude.^8^ All in all, this complexity underscores the necessity for nuanced interpretation of our RCT results and emphasizes the importance of additional control in future research. Finally, neither therapy completely eradicated PLP, suggesting that solely activating neural circuitry by PMI or PME may be insufficient to fully resolve PLP. Combining both methods or integrating somatosensory training could potentially enhance pain reduction.^2,26^

In conclusion, although numerous PLP treatments have been proposed, each motivated by different hypotheses, few have been tested in RCTs. Although our main finding was that PME was not superior to PMI, in the present study, we observed a substantial reduction in PLP, improvements in secondary outcomes, and no adverse effects through an international, multicenter, double-blind RCT. These results suggest that PME and PMI aided by XR are likely to produce a meaningful reduction of PLP in most patients. Therefore, both PMI and PME can be considered as viable treatment options for PLP as they present valid alternatives to pharmacological interventions.

Nonetheless, the negative result regarding our superiority hypothesis enphasizes the need for further research to explore the mechanisms behind PLP and to develop more targeted therapeutic approaches. Furthermore, several questions relevant for PLP management with this type of treatments remain: Could PMI be as effective with simpler equipment? Could a combination of PMI and PME yield better results, as suggested by sports science? Would combining PMI and PME with other approaches, like mirror therapy, lead to greater reductions in PLP? These are open questions that future rigorous RCTs should seek to answer, with essential collaborations among clinicians, researchers, and engineers for a comprehensive, patient-centered approach to PLP management.

## Conflict of interest statement

M.M.N. and S.I. were employed by Integrum AB during the execution of this study. M.O.C. and M.B.K. consulted for Integrum AB during the execution of this study. M.O.C. and S.I. own shares of Integrum AB. M.O.C. is inventor in a patent related to the technology used in this study, but has no ownership.

## Appendix A. Supplemental digital content

Supplemental digital content associated with this article can be found online at http://links.lww.com/PAIN/C156.
