# Supplementary material for: Extended reality used in the treatment of phantom limb pain: a multicenter, double-blind, randomized controlled trial
Source: Pain. 2024 Sep 5;166(3):571–86. doi: 10.1097/j.pain.0000000000003384 (PMC11808706; doi:10.1097/j.pain.0000000000003384)
Supplement: Supplementary file 1 [file jop-166-571-s001.pdf]

Supplementary Appendix

Table of contents

Table of Figures 1

Table of Tables 2

Extended Methods 3

Outcome measures 3

Statistical Analyses 6

Extended results 8

Supplementary Figures and Tables 11

References 51

Table of Figures

Figure S1: Analysis of associations between missingness of two variables. 13
Figure S2: Analysis of associations between the missingness of variables. 14

Figure S3: Correlating variables. 15

Figure S4: Example of Fisher's permutation test. 16

Figure S5: ANCOVA – Distribution of estimates and p-values 28

Figure S6: PRI trend (PP) 37

Figure S7: NRS PLP trend (ITT) 38

Figure S8: WPD trend (ITT) 39

Figure S9: NRS Residual Limb Pain trend (ITT) 40

Figure S10: NRS PM (ITT) 41

Figure S11: NRS PLS trend (ITT)........ 42

Figure S12: NRS Interference with sleep (ITT) 43

Figure S13: NRS Interference with activities of daily living (ITT) 44

Figure S14: NRS Interference with work (ITT)........ 45

Figure S15: Exponential Decay model........ 49

Table of Tables

Table S1: List of participating partners. 11

Table S2: Subject Disposition. 17

Table S3: Baseline characteristics of the Per Protocol population. 18

Table S4: Prior treatments 20

Table S5: Prior medication. 21

Table S6: Medication usage at baseline........ 22

Table S7: Medication usage of patients withdrawing from the study........ 24

Table S8: Medication usage in the FAS population. 25

Table S9: Descriptive summary of changes in medication consumption. 27

Table S10: Exploratory analyses. 29

Table S11: Analysis of dichotomous secondary and exploratory variables........ 35

Table S12: McGill pain descriptors throughout the study in the FAS population 46

Table S13: Frequency of PLP in the FAS population 48

Extended Methods

The list of participating partners is presented in Table S1**Fout! Verwijzingsbron niet gevonden.**. One clinical partner (Fysische Geneeskunde en Revalidatie University Hospital Gent, Belgium) listed in the published protocol (Lendaro et al., 2018), did not enroll any subjects and was subsequently excluded from the trial. Conversely, an investigational site was added to the study after the protocol article was published (Shirley Ryan Ability Lab, Chicago, IL, USA).

For details about the treatment programs, refer to Supplementary File 2 of the published clinical protocol (Lendaro et al., 2018).

Outcome measures

Before analysis, some outcome measures required additional steps to summarize them. In the following we briefly describe the measures and steps taken:

- Pain Rating Index (PRI): the sum of the scores for all descriptors of the Short Form of the McGill Pain Questionnaire (Melzack, 1987) was calculated. The PRI is a score ranging from 0 to 45. Higher scores indicate worse pain.
- Weighted Pain Distribution (WPD): the index is a study-specific questionnaire used in previous studies computed by adding together the different levels of pain experienced in a day (Lendaro et al., 2017; Ortiz-Catalan et al., 2014, 2016). Pain is rated on the present pain intensity scale (Melzack, 1975). The weighting factors are the portion of time spent in a given level, where 1 is 100% of the awake time. The WPD index ranges from 0 to 5. Higher scores indicate worse pain.
- Significant change in PRI: the number of patients who reached a clinically meaningful reduction in PLP (>50%) at end of treatment and follow-up assessments with respect to baseline.
- PLP intensity on the numeric rating scale (PLP NRS): PLP intensity evaluated with an integer score from 0 to 10. Higher scores indicate worse pain.
- Phantom Limb sensations on the NRS (PLS NRS): Pain limb sensation intensity evaluated with an integer score from 0 to 10. Higher scores indicate more vivid sensations.
- Residual Limb pain on the NRS (RLP NRS): Residual Limb pain intensity evaluated with an integer score from 0 to 10. Higher scores indicate worse pain.
- Phantom motor ability on NRS: the perceived ability to control phantom limb movements evaluated with an integer score from 0 to 10. Higher scores indicate better-perceived control.

- Telescoping: evaluated with a score from 0 to 6 evaluating the perceived length of the phantom limb in relation to the length of the real limb. 6 indicates no telescoping and 0 indicates the phantom limb completely telescoped into the residual limb.
- Pain Frequency: PLP frequency evaluated with one of 6 ordered frequency descriptors.
- The Present Pain Intensity (PPI): PLP evaluated with one of 6 ordered pain descriptors.
- PLP interference with work: It is evaluated on an NRS from 0 to 10. Higher scores indicate more interference.
- PLP interference with activities of daily living: It is evaluated on an NRS from 0 to 10. Higher scores indicate more interference.
- PLP interference with sleep: It is evaluated on an NRS from 0 to 10. Higher scores indicate more interference.
- EuroQol-5D-5L (EQ5D5L): the questionnaire results in a "health state" which is a 5-digit sequence composed of the scores of each question. The "health state" is a converted score using country-specific value sets. The ranges vary from country to country, however an index of 1 indicates full health and lower scores correspond to lower health states (Herdman et al., 2011). For this publication, the following value sets were used:
    - Irish value sets for Investigation Site 1 (NUI) (Hobbins et al., 2018)
    - Danish value sets[1] for Investigation Sites 2 (OUH),3 (RCS), and 6 (SUH) (Jensen et al., 2021)
    - German value sets from Investigation Site 4 (RUB) (Ludwig et al., 2018)
    - Hungarian value sets[Fout! Bladwijzer niet gedefinieerd.] for Investigation Site 9 (URI) (Rencz et a l., 2020)
    - Dutch value sets for Investigation Site 7 (UMG) (Versteegh et al., 2016)
    - Canadian value sets for Investigation Site 8 (UNB) (Xie et al., 2016)
    - American value sets for Investigation Site 5 (SRA) (Pickard et al., 2019)
- Pain Catastrophizing Scale-6 (PCS-6): the total score was calculated as the sum of scores for the six individual items and has a range from 0 to 24. Higher scores indicate worse catastrophizing (Sullivan et al., 1995).
- Pain Self-Efficacy Questionnaire-2 (PSEQ-2): the total score was calculated as the sum of scores for the two individual items and has a range from 0 to 12. Higher scores indicate better pain self-efficacy (Nicholas, 2007).

[1] Validated value sets specific for this country were not available at the time of the analyses. Published value sets from country geographically close were chosen instead.

- Pain Disability Index (PDI): the total score was calculated as the sum of scores for the seven individual items and has a range from 0 to 70. Higher scores indicate worse disability (Tait et al., 1990).
- The Patient Health Questionnaire-2 (PHQ-2): the total score was calculated as the sum of scores for the two individual items and ranges from 0 to 6. Higher scores correspond to more frequent depressed mood (Kroenke et al., 2001).
- Expectations for Complementary and Alternative Medicine Treatments (EXPECT): the questionnaire is composed of 4 items where the first question is used as a control to clarify the difference between hopes and expectations. The remaining three questions evaluate treatment expectations and are scored from 0 to 10. The mean of the score was taken. The questions were also analyzed separately (Jones et al., 2016).
- Health-Care Climate Questionnaire (HCCQ): the questionnaire is evaluated with 6 items rated from 0 to 7, which are summed together, with a range from 0 to 42. Higher scores indicate a more supportive interaction with the therapist (Williams et al., 1998).
- Opinion About Treatment (OAT): this 3-item questionnaire is scored from 1 to 9 per item. The total score is the sum of the single items and ranges from 3 to 27. Higher scores indicate a more supportive interaction with the therapist (Mooney et al., 2015).
- Patient Global Impression of Change (PGIC): a single question used to rate the belief about the efficacy of treatment on a seven-point scale, ranging from 'no change' to 'a great deal better' (Hurst & Bolton, 2004).

All outcome variables were computed based on the score at visit 0 minus the score at the relevant assessment point (visit 15, 1-, 3- and 6-month follow-up), apart from the PGIC which was evaluated solely at the end of the last treatment session. Between group differences were computed as outcome for PME minus outcome for PMI.

Depending on the specific variable, improvements were in some cases marked by an increase or in other cases by a decrease in the score. For this reason, signs associated with within-group and between-group differences need to be interpreted in the context of the specific variable.

Medications

Medications used prior to entering the trial and at the start of the trial were reported utilizing the 4th and 5th level of the Anatomical Therapeutic Chemical Classification System (ATC codes). Concomitant use of medications during the trial was described using the World Health Organization (WHO) Analgesic Ladder (Anekar & Cascella, 2022; World Health Organization, 2018). In short, the WHO analgesic ladder was a strategy proposed by the WHO to provide pain relief for cancer

patients. From when it was proposed in 1986, the WHO pain ladder has been modified and is currently applied also to acute and chronic non-cancer painful conditions. The ladder consists of three steps (Anekar & Cascella, 2022):

- First step (mild pain): non-opioid analgesics such as nonsteroidal anti-inflammatory drugs (NSAIDs) or acetaminophen with or without adjuvants
- Second step (moderate pain): weak opioids with or without non-opioid analgesics, and with or without adjuvants
- Third step (severe and persistent pain): strong opioids with or without non-opioid analgesics, and with or without adjuvants.

Adjuvant medications refer to a large group of drugs belonging to different classes which are typically administered for conditions other than pain. Adjuvants include, for example, antidepressants, anticonvulsants, topical anesthetic therapies, corticosteroids, bisphosphonates, and cannabinoids. However, adjuvants are also indicated as a first-line treatment option for specific neuropathic pain conditions (Attal et al., 2010).

To capture concomitant usage of different classes of medications and possible changes related to changes in pain levels throughout the treatment and follow up, we classified the specific medication regimen of each patient according to the three steps of WHO pain ladder or exclusive use of adjuvant medications. We reported this for Visit 0, Visit 15 and at the 3 follow-up assessments.

Statistical Analyses

Analysis of missingness of the data

The dataset was first analyzed to establish the type of missingness. Fisher's exact test was used to determine the significance of association between the missingness of two variables. The results of this correlation analysis are shown in Figure S1. We subsequently tested the significance of associations between the missingness of one variable and the value of all the other available variables. To carry out this analysis we used the Wilcoxon rank sum test for continuous/ordinal variables, and the Kruskal-Wallis test for categorical variables. The results of this analysis are shown in Figure S2. These analyses, and the consideration that participants who missed one visit would present all the outcomes recorded at that time point as missing, led us to conclude that the data in our database were Missing At Random (MAR).

Stochastic regression imputation of missing data

To carry out the statistical analyses of the outcome measures on the full ITT population, we imputed the missing data using stochastic regression. Briefly, we first identified possible covariates for the

stochastic regression imputation models by testing the magnitude and statistical significance of the associations between every variable and all the other variables and factors. Associations between ordinal/continuous variables and factors were quantified in terms of the Spearman's correlation coefficient. For associations between continuous variables and categorical factors we quantified significant differences in distributions across categories using the Kruskal-Wallis' test. The results of this analysis are shown in Figure S3.

For every missing data point we built a linear regression model choosing the significantly associated variables and factors (defined as $p \leq 0.01$ and Spearman's $\rho \geq 0.6$, if applicable) as covariates. Additionally, we fitted all the available data from patients receiving the same treatment (fixed factor) and used the distribution of residuals to randomly sample 50 values to impute. This led us to the creation of 50 new datasets for the analyses of between-group differences on the ITT population. This means that for a given outcome variable, a statistical test was carried out on each and every dataset and the 50 results were averaged together to yield the final result.

Statistical analyses

All the statistical comparisons using Fisher's permutation test and Fisher's Exact test were performed by randomly resampling the dataset 10,000 times to construct the null distribution of the differences between the two groups. For each outcome analysed with this methodology we obtained 50 results, which were then averaged together. We report the results as means over the 50 imputed datasets for within-group changes, between-group differences, treatment effect, p-value and the respective standard deviations. An example of the null distribution of differences in PRI and the observed mean for one dataset is shown in Figure S4. Note that the permutation test does not provide a confidence interval for the difference of means of the two groups (Hayes, 2020). Confidence with respect to the p-value is achieved through the high number of permutations. For this reason, in contrast to the published SAP, we do not report 95% confidence intervals. Effect sizes for Fisher's Permutation test were calculated with Cohen's D, odds ratios were calculated for Fisher's exact test, and Cramér's V was calculated for the Mantel-Haenszel chi square test.

Post-hoc Bayes Factor analysis

The Bayes factor is an expression of the empirical support for a certain hypothesis (Makin & Orban de Xivry, 2019; Morey et al., 2016). Bayes factor 10 formally indicates the ratio of the likelihood of the alternative hypothesis to the likelihood of the null hypothesis. Conversely, Bayes factor 01 indicates the support in favor of the null hypothesis over the alternative. In the present study, we performed a post-hoc (not included in the SAP) Bayes factor 10 analysis for $\mu_{PME}>\mu_{PMI}$. We decided to perform this analysis using the parameters from the ANOVA test described in the sensitivity

analyses, which was performed for the ITT population as this allowed us to use a MATLAB package for Bayes Factor analysis freely available (Krekelberg, 2022). We calculated a Bayes factor for each of the 50 ANCOVA tests required for the ITT population and computed the mean and standard deviation of the 50 factors obtained. The interpretation of Bayes Factor that was adopted in the current work was proposed by (Andraszewicz et al., 2015).

Extended results

The subject disposition and definition of intent-to-treat (ITT) population, Full Analysis Set (FAS) and per protocol (PP) populations are presented in Table S2, which reports also the details of the protocol deviations.

Baseline characteristics for the ITT population are reported in the manuscript. Baseline characteristics for the PP population are reported in Table S3. History of previous treatments and medications for PLP are reported Table S4 and Table S5.

Concomitant use of medications during the study was allowed and recorded. The complete list of medications in use at baseline is reported in Table S6. For this table, it is important to note that most patients in this study made simultaneous use of multiple medications. Overall, the most commonly used drugs were amitriptyline, pregabalin, gabapentin and paracetamol. Additionally, the control group of this study registered a more frequent usage of oxycodone (strong opioid) at baseline.

To get a detailed picture of drug usage, we classified the specific medication regimen of each patient according to the WHO analgesic ladder or as exclusive use of adjuvants at Visit 0, Visit 15 and at the 3 follow-up assessments. Since the objective in tracking medication consumption was merely to inform the interpretation of the results, we did not predefine a specific strategy to account for missing data (e.g. subjects that discontinued their participation to the study). It is, however, important to consider the medication use of participants who prematurely discontinued the trial. In Table S7 we report the details of the medication usage of those participants who did not complete the study. Specifically, we show medication usage for the FAS population Table S8. Here, only the participants still enrolled in the study were considered and percentages are reported with respect to the available participants. This way of reporting does not consider that participants withdrawing from the study might have consumed medications and the percentages of medication users at any given assessment point can be used to draw comparisons.

We believe that this is the most accurate way to represent data affected by missingness that was not imputed with our stochastic regression model, such as in this case.

At baseline, a larger proportion of participants in the control group consumed medications and a significantly larger proportion of patient in the control group was on the highest level of the WHO analgesic pain ladder (25.9% vs 13.5%), indicating a more common use of strong opioids in the control group. Throughout the treatment, no significant group differences in medication usage emerged between the two groups. Overall changes in overall medication usage in the two groups can be primarily reconducted to individuals withdrawing from the study. In a few participants, changes in dosage were observed and a descriptive summary is reported in Table S9. Most of the changes involved adjuvant medications.

All the results from the analyses of primary and secondary outcomes in the ITT population and PP population, together with the sensitivity analyses, are reported in the manuscript. Figure S5 additionally reports the histograms of the p-values and estimates for the adjusted comparison of the primary outcome (ANCOVA). Exploratory outcome analyses on the ITT population are reported in Table S10.

For completeness, we report the trends for all the variables that have been used to assess PLP (PRI, WPD, NRS) and the ratings for phantom sensations (PLS), telescoping, phantom movements (PM) and residual limb pain (RLP) (Figure S6 through Figure S13). In particular, Figure S6 illustrates the trend of PRI scores (the primary outcome variable) for the per-protocol (PP) population. This graph offers valuable insights into the qualitative impact of imputation on preserving the integrity of the data distribution. As is evident from the figure, the imputation process does not result in any significant distortion.

Among the secondary and exploratory outcome variables the following were analyzed as dichotomous outcomes: presence of clinically significant reduction of PRI (PRI reduction > 50%), telescoping, and PLP frequency. Results of these analyses are summarized in Table S11.

The PRI index is computed as the sum of the individual scores to the pain descriptors in the short form of the McGill questionnaire (Melzack, 1987) and the change in usage of the pain descriptors is summarized in Table S12 for both treatment and follow up. Details on the frequency of PLP are given in Table S13.

Figure S15 shows the comparison of the PRI trend from Visit 0 to Visit 15 for the two groups fitted to an exponential decay model for both the ITT and the PP population. The results of this analysis seem to suggest that the PME group experienced an improvement in pain at a faster rate in the first 2-3 treatment sessions. The advantage of the PME group disappears after session 4-5. It could be speculated that the PMI group had initially a slower improvement in pain due to the well-known

individual differences in the ability to perform motor imagery (Collet et al., 2011). Some participants in the control group might have taken longer to identify a strategy to comply with the instructions from the therapists while at the same time not performing actual motor execution. On the other hand, motor execution might be more intuitive and straightforward to perform for a newcomer, this might have allowed participants in the PME group to progress faster.

We ran a post hoc Bayesian analysis of the result of the ANCOVA test to quantify the evidence in support of the alternative hypothesis. However, given that the result estimated by the ANCOVA test showed $improvement_{PMI}$>$improvement_{PME}$, we eventually calculated the Bayes factor 10 post-hoc for H2: $improvement_{PMI}$>$improvement_{PME}$, thus aligning the calculation to the observed data. In this case, the Bayes factor 10 was found to be 0.13±0.01 which indicates that the evidence in support of the null hypothesis (no difference between the two groups) is substantial and roughly 10 times stronger than the evidence in support of H2: $improvement_{PMI}$>$improvement_{PME}$.

Our study allows patients to choose the frequency of their treatment sessions, intending to improve adherence and accommodate scheduling capabilities across multiple sites. Based on our previous studies, we hypothesized that the cumulative amount of therapy received, rather than the frequency or specific timing of treatment, would be the most significant factor in improving patient outcomes.

Further, in order to effectively engage with these therapies, patients needed to learn how to effectively control the VR via myoelectric pattern recognition for PME, or how carry out motor mental visualization in complete relaxation for PMI. This learning, unfolding over several sessions, highlights that therapeutic benefits are deeply influenced by patients' active learning and adaptation to the therapy. Therefore, evaluating outcomes against a specific post-randomization timeline might overlook the significance of cumulative therapy exposure necessary for learning, potentially rendering comparisons less equitable. To rigorously assess the potential impact of treatment frequency on outcomes, we have now conducted a subgroup analysis to determine whether the chosen frequency of therapy sessions influenced the results (see Figure S16). Our analysis demonstrates no noteworthy differences in the PRI among the subgroups, yet this is a post-hoc analysis and a more thorough investigation is needed to explore the effect of frequency on outcomes.

Supplementary Figures and Tables

Table S1: List of participating partners.

The trial Study Group (Site Principal Investigators noted):
**Coordinating group**<br>Chalmers University of Technology, Gothenburg, Sweden: Max Ortiz Catalan (PI), Eva Lendaro (I/Monitor)
**Investigation Site 1 (NUI)**<br>Centre for Pain Research, National University of Ireland, (NUI) Galway, Ireland: Brian McGuire (PI), Paul O'Reilly
**Investigation Site 2 (OUH)**<br>Örebro University Hospital (OUH), Örebro, Sweden: Liselotte Hermansson (PI), Cathrine Widehammar, Karin Eriksson, Nicholas Ståhl Haldosen
**Investigation Site 3 (RCS)**<br>Rehabcenter Sfären, Bräcke Diakoni (RCS), Stockholm, Sweden: Anita Stockselius (PI), Lena Gudmundson
**Investigation Site 4 (RUB)**<br>Department of Psychosomatic Medicine and Psychotherapy, LWL University Hospital, Ruhr University Bochum (RUB), Bochum, Germany: Martin Diers (PI), Larissa Cordier, Martin Bordewieck, Laura Katona
**Investigation Site 5 (SRA)**<br>Shirley Ryan Ability Lab (SRA), Chicago, IL, USA: Levi Hargrove (PI), Kristi Turner, Andrea Ikeda

Investigation Site 6 (SUH)

Sahlgrenska University Hospital (SUH), Gothenburg, Sweden: Lina Bunketorp Käll (PI), Maria Munoz, Ingrid Rignér, Emilia Diamantidis, Katarzyna Kulbacka-Ortiz, Nena Segerbrand

Investigation Site 7 (UMG)

University Medical Center Groningen (UMG), Groningen, Netherlands: Corry K. van der Sluis (PI), Els Keesom, O van der Niet, PA Wijdenes, M van der Groep, SH de Jong

Investigation Site 8 (UNB)

Atlantic Clinic for Upper Limb Prosthetics, Institute of Biomedical Engineering, University of New Brunswick, (UNB) New Brunswick, Canada: Wendy Hill(PI), Kristel Desjardins

Investigation Site 9 (URI)

University Rehabilitation Institute (URI), Ljubljana, Slovenia: Helena Burger(PI), Zdenka Pihlar, Tonja Robida, Metka Zalar

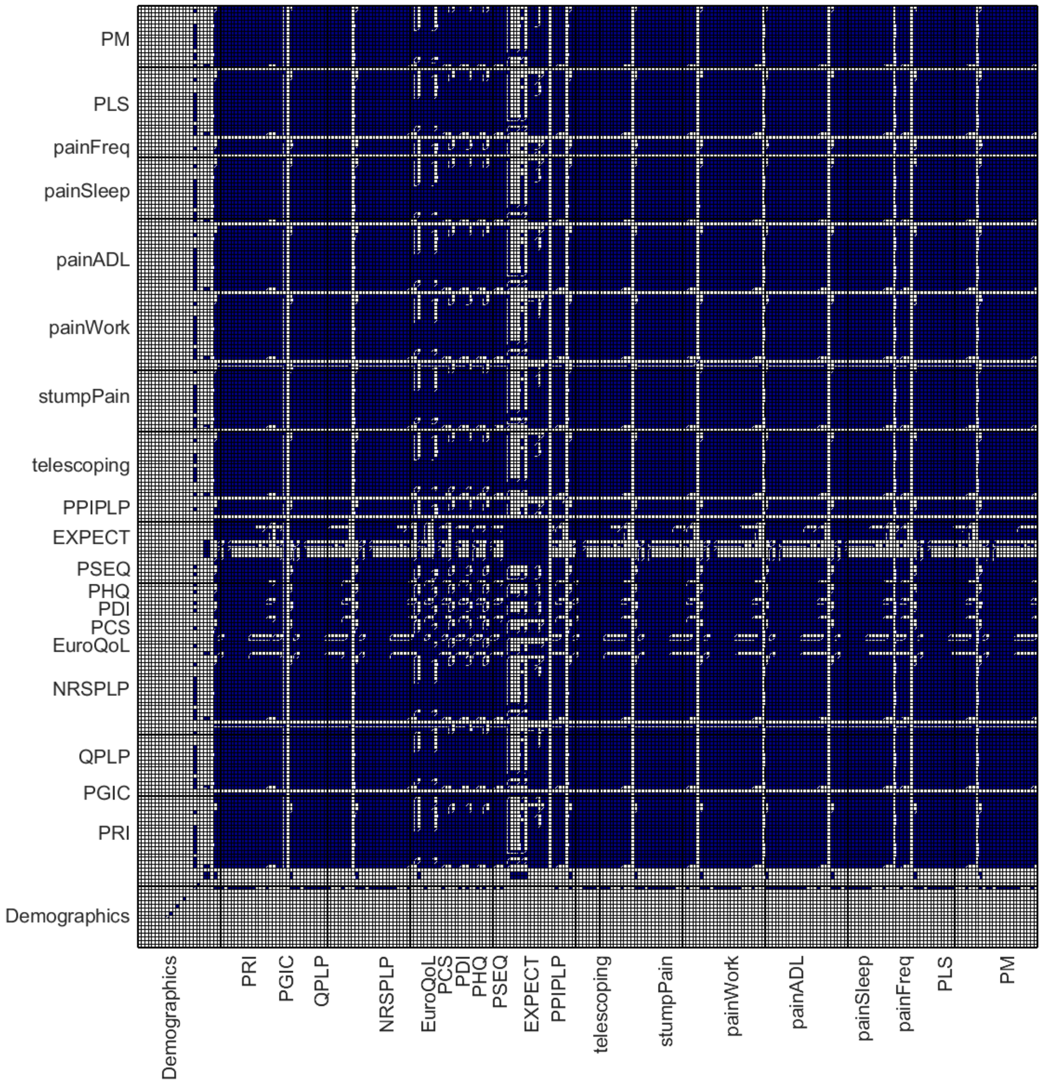

Figure S1: Analysis of associations between missingness of two variables.

Blue indicates significant associations (p<0.05), no correction for multiple comparisons was applied. The patterns of association highlight that subjects that missed one visit were likely to present all the other outcomes recorded at that time point as missing. Further, participants who missed one visit were more likely to miss subsequent visits.

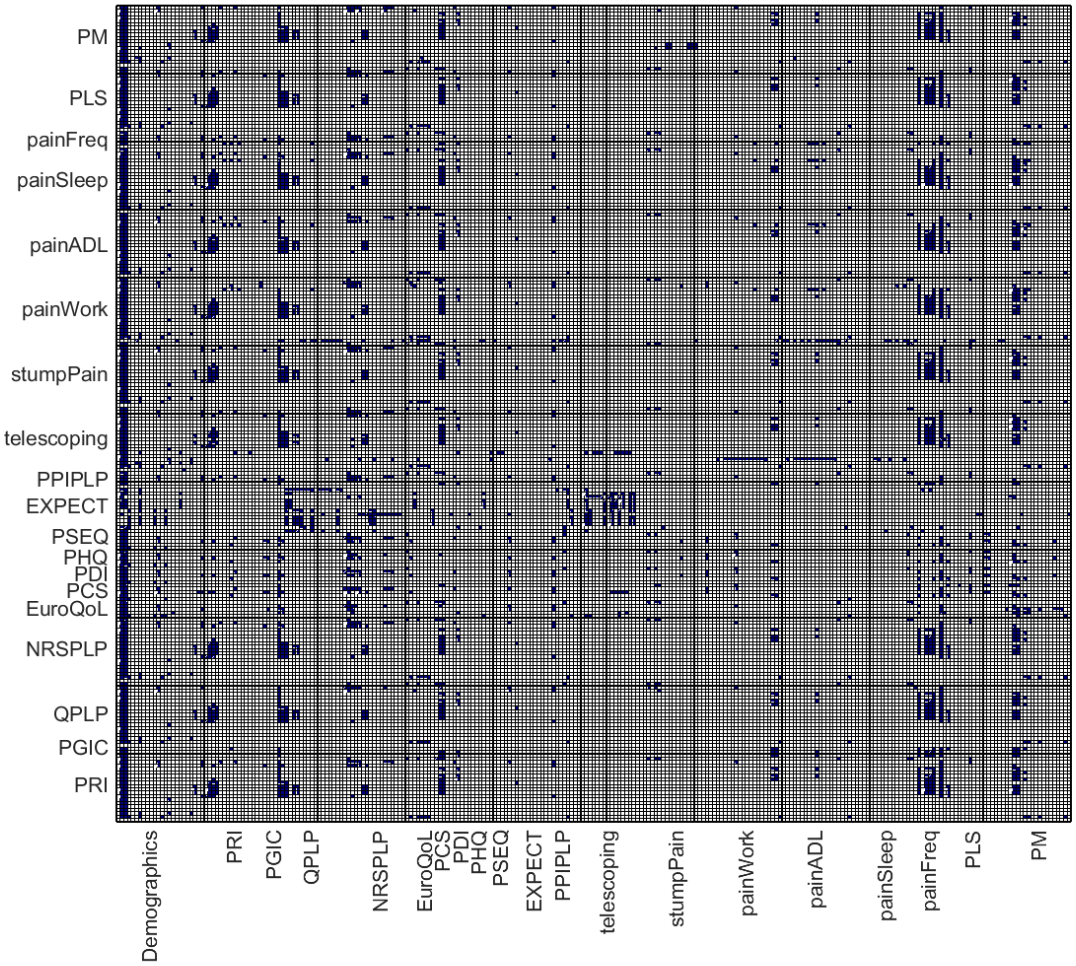

Figure S2: Analysis of associations between the missingness of variables.

Missingness of one variable correlated to the missingness of other variables. Blue indicates significant associations (p<0.05), no correction for multiple comparisons was applied. From the plot, it is possible to notice a lack of correlation between a certain missing data point and the available values of the same outcome measure (lack of correlations on the diagonals) supporting the conclusion that the data are missing at random.

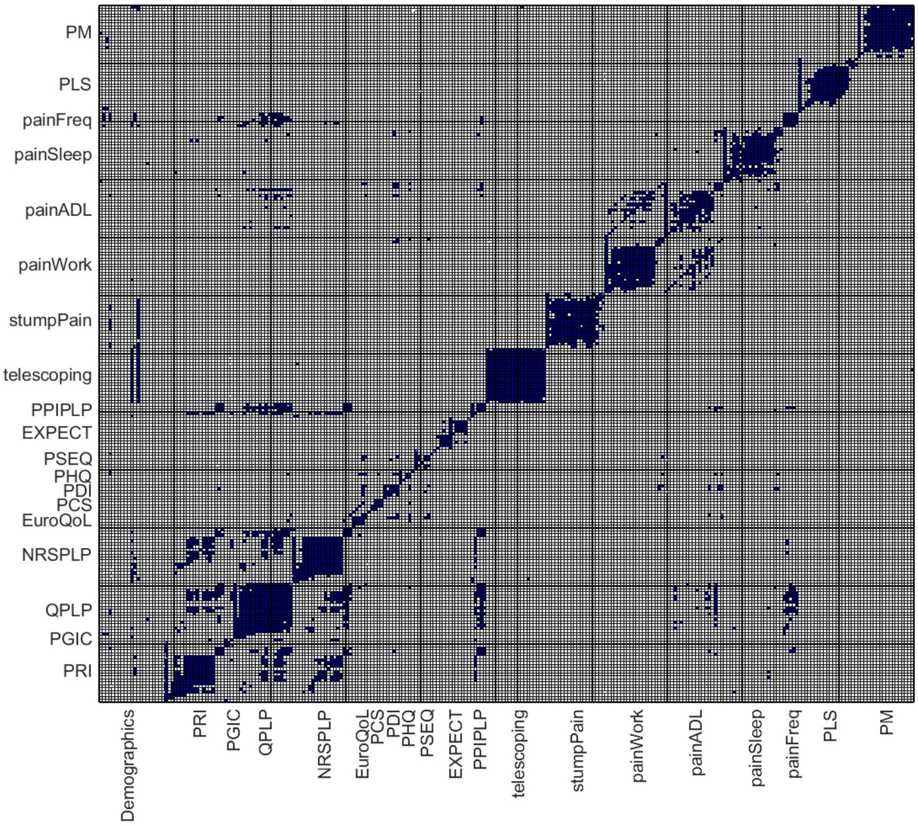

Figure S3: Correlating variables.

Variables that were identified as significantly correlated among the available ones were selected as covariates for the stochastic regression imputation procedure.

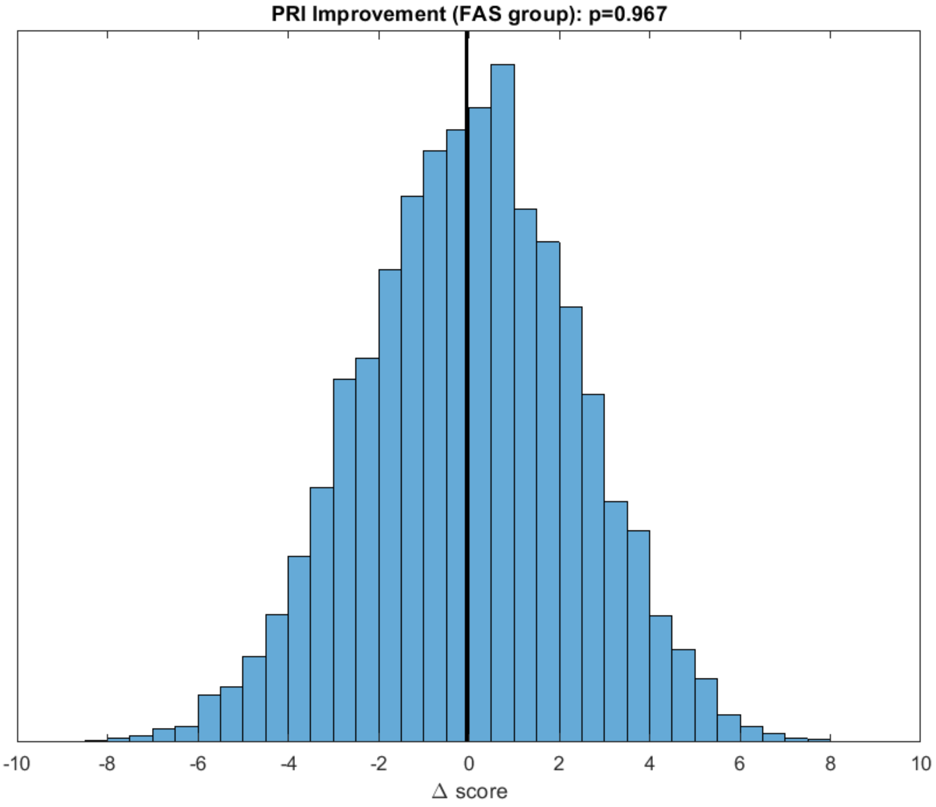

Figure S4: Example of Fisher's permutation test.

The histogram represents the distribution of the null hypothesis for PRI differences between the two treatments. The null distribution is constructed by randomly resampling the given dataset. The process is repeated 10,000 times yielding 10,000 possible differences in PRI decrease between two groups randomly shuffled. The example is taken from the analysis of changes in PRI in the FAS population where the black line indicates the observed difference in pain change between PME and PMI which in this case is -0.06 and associated p-value of 0.967. The negative sign indicates a larger improvement in the PMI group. In the case of ITT analyses, this process is repeated for every imputed dataset. In this study, we used 50 imputed datasets. Abbreviations: PRI, Pain Rating Index; FAS, Full Analysis Set: ITT, Intent To Treat

Table S2: Subject Disposition.

Subject Disposition by Treatment Group			
	Overall	*PME*	*PMI*
A. Total screened subjects	*145*	*N.A.*	*N.A.*
1. Did not meet inclusion criteria	*43*	*N.A.*	*N.A.*
2. Declined	*12*	*N.A.*	*N.A.*
3. Other reasons for exclusion	*9*	*N.A.*	*N.A.*
B. Subjects randomized	*81*	*53*	*28*
C. Missing complete data	*1*	*1*	*0*
D. Protocol Deviations	*25*	*14*	*11*
1. Did not receive allocated intervention	*6*	*3*	*3*
2. Discontinued intervention	*11*	*8*	*3*
3. Received fewer than 15 treatment sessions	*7*	*3*	*4*
4. Blinding failure	*1*	*0*	*1*
ITT population (B-C)	*80*	*52*	*28*
FAS population (B- C - D1 - D2)	*63*	*41*	*22*
PP population (B - C -D)	*55*	*38*	*17*

The table includes the reasons for exclusion from the study of screened participants, along with the reasons for protocol deviations and exclusion from PP population. A) total number of participants that were considered for participation in the study; B) number of participants that underwent Visit 0 and were assigned to a treatment group; C) number of participants whose data was unretrievably lost; D) number of participants with protocol deviations, 1: discontinued the trial before taking part to any treatment session, 2: ended their participation to the trial prematurely and did not complete 15 treatment sessions, 3: completed Visit 15 but attended fewer than 15 treatment sessions, 4: the evaluator was not blinded to treatment allocation. Abbreviations: PME, Phantom Motor Execution; PMI, Phantom Motor Imagery; PP, Per Protocol; ITT, Intent-to-treat; FAS, Full Analysis Set

Table S3: Baseline characteristics of the Per Protocol population.

Baseline Characteristics to Be Reported by Treatment Group (PP population)					
n(%) unless otherwise specified			***PME (n=37)***	***PMI (n=17)***	***Overall (n=54)***
Background information					
Age at randomization [years, mean (SD)]			*57.89 (13.97)*	*59.41 (9.38)*	*58.37 (12.64)*
Height [cm, mean (SD)]			*171.81 (16.83)*	*172.88 (17.17)*	*172.15 (16.78)*
Weight [kg, mean (SD)]			*81.57 (17.82)*	*83.24 (20.14)*	*82.09 (18.41)*
Sex	*Females*		*9 (24.32%)*	*7 (41.18%)*	*16 (29.63%)*
	Males		*28 (75.68%)*	*10 (58.82%)*	*38 (70.37%)*
Time since amputation [years, median (IRQ)]			*5.58 (2.75-10.33)*	*9.67 (5-18.67)*	*5.08 (2.25-10.83)*
Time since onset of PLP [years, median (IRQ)]			*4.92 (1.83-10.33)*	*7.58 (3.44-15.04)*	*7.29 (2.85-13.83)*
Reason for amputation	*trauma*		*21 (56.76%)*	*5 (29.41%)*	*26 (48.15%)*
	cardiovascular		*10 (27.03%)*	*4 (23.53%)*	*14 (25.93%)*
	cancer		*4 (10.81%)*	*4 (23.53%)*	*8 (14.81%)*
	diabetes		*1 (2.7%)*	*1 (5.88%)*	*2 (3.7%)*
	other		*1 (2.7%)*	*3 (17.65%)*	*4 (7.41%)*
Type of prosthesis	*none*		*10 (27.03%)*	*6 (35.29%)*	*16 (29.63%)*
	active/myoelectric		*9 (24.32%)*	*2 (11.76%)*	*11 (20.37%)*
	cosmetic		*2 (5.41%)*	*0 (0%)*	*2 (3.7%)*
	passive		*12 (32.43%)*	*8 (47.06%)*	*20 (37.04%)*
	body-powered		*4 (10.81%)*	*1 (5.88%)*	*5 (9.26%)*
Time with current prosthesis (among users) [months, median (IRQ)]			*18 (4.5-30)*	*54 (24-60)*	*24 (11.25-54)*
Prosthesis usage (among users) [hours, median (IRQ)]			*12 (9-14)*	*9 (5-14.5)*	*12 (7.25-14)*
Telescoping, yes			*17 (45.95%)*	*10 (58.82%)*	*27 (50%)*
Minimization factors	*Level of amputation*	*transfemoral*	*19 (51.35%)*	*8 (47.06%)*	*27 (50%)*
		transtibial	*10 (27.03%)*	*6 (35.29%)*	*16 (29.63%)*
		transmetatarsal	*0 (0%)*	*0 (0%)*	*0 (0%)*
		transhumeral	*6 (16.22%)*	*2 (11.76%)*	*8 (14.81%)*
		transradial	*2 (5.41%)*	*1 (5.88%)*	*3 (5.56%)*

		transmetacarpal	*0 (0%)*	*0 (0%)*	*0 (0%)*
	Intensity of PLP	*high (> 4)*	*23 (62.16%)*	*10 (58.82%)*	*33 (61.11%)*
		low (<= 4)	*14 (37.84%)*	*7 (41.18%)*	*21 (38.89%)*
	Investigational site	*#1*	*2 (5.41%)*	*1 (5.88%)*	*3 (5.56%)*
		#2	*3 (8.11%)*	*2 (11.76%)*	*5 (9.26%)*
		#3	*5 (13.51%)*	*2 (11.76%)*	*7 (12.96%)*
		#4	*3 (8.11%)*	*1 (5.88%)*	*4 (7.41%)*
		#5	*9 (24.32%)*	*3 (17.65%)*	*12 (22.22%)*
		#6	*12 (32.43%)*	*7 (41.18%)*	*19 (35.19%)*
		#7	*2 (5.41%)*	*0 (0%)*	*2 (3.7%)*
		#8	*1 (2.7%)*	*1 (5.88%)*	*2 (3.7%)*
Chosen treatment frequency	*once a week*		*6 (16.22%)*	*3 (17.65%)*	*9 (16.67%)*
	twice a week		*20 (54.05%)*	*9 (52.94%)*	*29 (53.7%)*
	five times a week		*11 (29.73%)*	*5 (29.41%)*	*16 (29.63%)*
Intensity of PLP [NRS(0-10), mean (SD)]			*4.76 (2.42)*	*4.59 (2.81)*	*4.7 (2.52)*
Intensity of residual limb pain [NRS(0-10), mean (SD)]			*1.35 (2.12)*	*1.24 (2.31)*	*1.31 (2.52)*
Intensity of PLS [NRS(0-10), mean (SD)]			*5.19 (3.65)*	*5 (3.79)*	*5.13 (3.66)*
Intensity of voluntary phantom movements [NRS(0-10), mean (SD)]			*5.97 (3.44)*	*7.94 (2.25)*	*6.59 (3.23)*
Pain Rating Index at baseline [PRI (0-60), mean (SD)]			*15.03 (7.86)*	*14.94 (7.61)*	*15 (7.71)*
Pain interference with sleep [NRS(0-10), mean (SD)]:			*4.59 (3.74)*	*6.18 (3.34)*	*5.09 (3.66)*
Pain interference with daily life activities [NRS(0-10), mean (SD)]:			*4.14 (2.98)*	*4.53 (3.52)*	*4.26 (3.13)*
Pain interference with work [NRS(0-10), mean (SD)]:			*3.53 (2.82)*	*3.27 (3.71)*	*3.45 (3.07)*
Presence of telescoping [NRS(0-10), mean (SD)]			*4.53 (1.78)*	*4.47 (1.62)*	*4.51 (1.72)*
Weighted Pain Distribution Index [WPD(0-5), mean (SD)]:			*1.95 (1.08)*	*1.94 (1.34)*	*1.95 (1.15)*

Abbreviations: PME, Phantom Motor Execution; PMI, Phantom Motor Imagery; IQR, Interquartile Range; NRS, Numeric Rating Scale; PP, Per Protocol

Table S4: Prior treatments

Patients with prior treatment			
Treatment type	***Overall (n=80)***	***PME (n=52)***	***PMI (n=28)***
Mirror therapy	*29 (36.25%)*	*19 (36.54%)*	*10 (35.71%)*
Acupuncture	*14 (17.5%)*	*12 (23.08%)*	*2 (7.14%)*
TENS	*18 (22.5%)*	*12 (23.08%)*	*6 (21.43%)*
Spinal cord stimulation	*3 (3.75%)*	*2 (3.85%)*	*1 (3.57%)*
Spinal Anaesthesia	*1 (1.25%)*	*1 (1.92%)*	*0 (0%)*
Nerve blockade	*3 (3.75%)*	*1 (1.92%)*	*2 (7.14%)*
Graded Motor Imagery	*2 (2.5%)*	*1 (1.92%)*	*1 (3.57%)*
EMDR	*5 (6.25%)*	*3 (5.77%)*	*2 (7.14%)*
Hypnotherapy/meditation	*4 (5%)*	*3 (5.77%)*	*1 (3.57%)*
Neuroma surgery	*1 (1.25%)*	*0 (0%)*	*1 (3.57%)*
Other experimental non-pharmacological treatments	*3 (3.75%)*	*2 (3.85%)*	*1 (3.57%)*
Psychotherapy	*1 (1.25%)*	*1 (1.92%)*	*0 (0%)*
Laser therapy	*1 (1.25%)*	*0 (0%)*	*1 (3.57%)*
Massage	*1 (1.25%)*	*0 (0%)*	*1 (3.57%)*
Cryotherapy	*1 (1.25%)*	*1 (1.92%)*	*0 (0%)*

List of treatments that participants underwent and completed? prior to being enrolled to the trial. Abbreviation: EMDR, Eye Movement Desensitization & Reprocessing; TENS, Transcutaneous Electrical Neurostimulation; PME, Phantom Motor Execution; PMI, Phantom Motor Imagery.

Table S5: Prior medication.

Patients with prior use of drug				
ATC Code 4th level	***ATC Code 5th level***	***Overall (n=80)***	***PME (n=52)***	***PMI (n=28)***
A07EA Corticosteroids acting locally	*A07EA03 Prednisone*	*1 (1.25%)*	*1 (1.92%)*	*0 (0%)*
M02AB Capsaicin and similar agents	*M02AB01 capsaicin*	*1 (1.25%)*	*1 (1.92%)*	*0 (0%)*
N02AA Natural opium alkaloids	*N02AA01 morphine*	*2 (2.5%)*	*2 (3.85%)*	*0 (0%)*
N02AE Oripavine derivatives	*N02AE01 buprenorphine*	*1 (1.25%)*	*0 (0%)*	*1 (3.57%)*
N02AJ Opioids in combination with non-opioid analgesics	*N02AJ06 codeine and paracetamol*	*2 (2.5%)*	*2 (3.85%)*	*0 (0%)*
N02AX Other opioids	*N02AX02 tramadol*	*1 (1.25%)*	*1 (1.92%)*	*0 (0%)*
N02BE Anilides	*N02BE01 paracetamol*	*2 (2.5%)*	*2 (3.85%)*	*0 (0%)*
N02BG Other analgesics and antipyretics	*N02BG10 cannabinoids*	*1 (1.25%)*	*1 (1.92%)*	*0 (0%)*
N03AE Benzodiazepine derivatives	*N03AE01 clonazepam*	*1 (1.25%)*	*0 (0%)*	*1 (3.57%)*
N03AF Carboxamide derivatives	*N03AF01 carbamazepine*	*1 (1.25%)*	*0 (0%)*	*1 (3.57%)*
N03AX Other antiepileptics	*N03AX16 pregabalin*	*4 (5%)*	*3 (5.77%)*	*1 (3.57%)*
	N03AX12 gabapentin	*4 (5%)*	*4 (7.69%)*	*0 (0%)*
N05BA Benzodiazepine derivatives	*N05BA01 diazepam*	*1 (1.25%)*	*1 (1.92%)*	*0 (0%)*
N06AA Non-selective monoamine reuptake inhibitors	*N06AA09 amitriptyline*	*2 (2.5%)*	*1 (1.92%)*	*1 (3.57%)*

List of medications that participants consumed and completed prior to being enrolled to the trial. Abbreviations: PME, Phantom Motor Execution; PMI, Phantom Motor Imagery.

Table S6: Medication usage at baseline.

Patients with concomitant use of drugs at visit 0				
ATC Code 4th level	***ATC Code 5th level***	***Overall (n=80)***	***PME (n=52)***	***PMI (n=28)***
A02BC Proton pump inhibitors	*A02BC01 omeprazole*	*4 (5%)*	*2 (3.8%)*	*2 (7.1%)*
M01AB Acetic acid derivatives and related substances	*M01AB05 diclofenac*	*2 (2.5%)*	*1 (1.9%)*	*1 (3.6%)*
M01AE Propionic acid derivatives	*M01AE01 ibuprofen*	*1 (1.3%)*	*1 (1.9%)*	*0 (0%)*
M03BX Other centrally acting agents	*M03BX01 baclofen*	*3 (3.8%)*	*1 (1.9%)*	*2 (7.1%)*
N02AA Natural opium alkaloids	*N02AA01 morphine*	*3 (3.75%)*	*1 (1.9%)*	*2 (7.1%)*
	N02AA05 oxycodone	*10 (12.5%)*	*5 (9.6%)*	*5 (17.9%)*
	N02AA59 codeine, combinations excl. Psycholeptics	*1 (1.3%)*	*0 (0%)*	*1 (3.6%)*
N02AB Phenylpiperidine derivatives	*N02AB03 fentanyl*	*3 (3.8%)*	*2 (3.85%)*	*1 (3.6%)*
N02AJ Opioids in combination with non-opioid analgesics	*N02AJ06 codeine and paracetamol*	*2 (2.5%)*	*1 (1.9%)*	*0 (0%)*
	N02AJ09 codeine and other non-opioid analgesics	*2 (2.5%)*	*2 (3.8%)*	*0 (0%)*
N02AX Other opioids	*N02AX02 tramadol*	*4 (5%)*	*4 (7.7%)*	*0 (0%)*
	N02AX06 tapentadol	*2 (2.5%)*	*1 (2.04%)*	*1 (3.6%)*
N02BA Salicylic acid and derivatives	*N02BA01 acetylsalicylic acid*	*1 (1.3%)*	*1 (1.9%)*	*0 (0%)*
N02BE Anilides	*N02BE01 paracetamol*	*15 (18.8%)*	*9 (17.3%)*	*6 (21.4%)*
N02BG Other analgesics and antipyretics	*N02BG10 cannabinoids*	*4 (5%)*	*4 (7.7%)*	*0 (0%)*
N03AX Other antiepileptics	*N03AX12 gabapentin*	*12 (15%)*	*5 (9.6%)*	*7 (25%)*
	N03AX16 pregabalin	*18 (22.5%)*	*14 (26.9%)*	*4 (14.3%)*
N05AH Diazepines, oxazepines, thiazepines and oxepines	*N05AH04 quetiapine*	*1 (1.3%)*	*1 (1.9%)*	*0 (0%)*

N03AE Benzodiazepine derivatives	*N05CD07 temazepam*	*1 (1.3%)*	*0 (0%)*	*1 (3.6%)*
	N05CD08 midazolam	*1 (1.3%)*	*0 (0%)*	*1 (3.6%)*
N05CF Benzodiazepine related drugs	*N05CF01 zopiclone*	*1 (1.3%)*	*1 (1.9%)*	*0 (0%)*
N06AA Non-selective monoamine reuptake inhibitors	*N06AA09 amitriptyline*	*15 (18.8%)*	*8 (15.4%)*	*7 (25%)*
	N06AA10 nortriptyline	*2 (2.5%)*	*2 (3.8%)*	*0 (0%)*
N06AB Selective serotonin reuptake inhibitors	*N06AB05 paroxetine*	*1 (1.3%)*	*0 (0%)*	*1 (3.6%)*
	N06AB10 escitalopram	*1 (1.3%)*	*1 (1.9%)*	*0 (0%)*
N06AX Other antidepressants	*N06AX05 trazodone*	*1 (1.3%)*	*0 (0%)*	*1 (3.6%)*
	N06AX21 duloxetine	*3 (3.8%)*	*1 (2.04%)*	*2 (7.1%)*
	N06AX26 vortioxetine	*1 (1.3%)*	*0 (0%)*	*1 (3.6%)*
N07BC Drugs used in opioid dependence	*N07BC51 buprenorphine, combinations*	*1 (1.3%)*	*1 (1.9%)*	*0 (0%)*
	N07BC02 methadone	*2 (2.5%)*	*1 (1.9%)*	*1 (3.6%)*
R05DA Opium alkaloids and derivatives	*R05DA03 hydrocodone*	*1 (1.3%)*	*1 (1.9%)*	*0 (0%)*
	R05DA04 codeine	*1 (1.3%)*	*1 (1.9%)*	*0 (0%)*

List of medications in use by the participants upon enrollment to the trial. Abbreviations: ATC code, Anatomical Therapeutic Chemical code; PME, Phantom Motor Execution; PMI, Phantom Motor Imagery.

Table S7: Medication usage of patients withdrawing from the study.

Medication usage in dropout participants				
		Overall (n=80)	***PME (n=52)***	***PMI (n=28)***
No medications	***v15***	*4 (5%)*	*3 (5.77%)*	*1 (3.57%)*
	1m	*5 (6.25%)*	*4 (7.69%)*	*1 (3.57%)*
	3m	*6 (7.5%)*	*5 (9.62%)*	*1 (3.57%)*
	6m	*6 (7.5%)*	*5 (9.62%)*	*1 (3.57%)*
Adjuvants	***v15***	*5 (6.25%)*	*3 (5.77%)*	*2 (7.14%)*
	1m	*5 (6.25%)*	*3 (5.77%)*	*2 (7.14%)*
	3m	*6 (7.5%)*	*3 (5.77%)*	*3 (10.71%)*
	6m	*9 (11.25%)*	*5 (9.62%)*	*4 (14.29%)*
Step 1 *(non-opioid + optional adjuvants)*	***v15***	*3 (3.75%)*	*2 (3.85%)*	*1 (3.57%)*
	1m	*3 (3.75%)*	*2 (3.85%)*	*1 (3.57%)*
	3m	*4 (5%)*	*3 (5.77%)*	*1 (3.57%)*
	6m	*2 (2.5%)*	*1 (1.92%)*	*1 (3.57%)*
Step 2 *(weak opioid + non-opioid + optional adjuvants)*	***v15***	*1 (1.25%)*	*1 (1.92%)*	*0 (0%)*
	1m	*0 (0%)*	*0 (0%)*	*0 (0%)*
	3m	*1 (1.25%)*	*1 (1.92%)*	*0 (0%)*
	6m	*2 (2.5%)*	*2 (3.85%)*	*0 (0%)*
Step 3 *(strong opioid + non-opioid + optional adjuvants)*	***v15***	*4 (5%)*	*2 (3.85%)*	*2 (7.14%)*
	1m	*3 (3.75%)*	*1 (1.92%)*	*2 (7.14%)*
	3m	*4 (5%)*	*1 (1.92%)*	*3 (10.71%)*
	6m	*4 (5%)*	*1 (1.92%)*	*3 (10.71%)*

Medication usage of participants withdrawing from the study prematurely. Data is shown for each assessment point. All numbers and percentages refer to the population at baseline. To note, some participants participated to the follow up assessments even though they prematurely ended the treatment, completing fewer than 15 sessions.

Abbreviations: PME, Phantom Motor Execution; PMI, Phantom Motor Imagery

Table S8: Medication usage in the FAS population.

Medication use FAS							
		Overall (n=80)		***PME (n=52)***		***PMI (n=28)***	
			%change		*%change*		*%change*
No medications	***v0***	*22 (27.5%)*		*16 (30.8%)*		*6 (21.4%)*	
	v15	*19 (30.2%)*	*2.7%*	*14 (34.1%)*	*3.4%*	*5 (22.7%)*	*1.3%*
	1m	*18 (28.1%)*	*-2%*	*13 (31%)*	*-3.2%*	*5 (22.7%)*	*0%*
	3m	*17 (28.8%)*	*0.7%*	*12 (30.8%)*	*-0.2%*	*5 (25%)*	*2.3%*
	6m	*18 (31.6%)*	*2.8%*	*12 (31.6%)*	*0.8%*	*6 (31.6%)*	*6.6%*
Adjuvants	***v0***	*25 (31.3%)*		*16 (30.8%)*		*9 (32.1%)*	
	v15	*18 (28.6%)*	*-2.7%*	*12 (29.3%)*	*-1.5%*	*6 (27.3%)*	*-4.9%*
	1m	*18 (28.1%)*	*-3.1%*	*12 (28.6%)*	*-2.2%*	*6 (27.3%)*	*-4.9%*
	3m	*17 (28.8%)*	*-2.4%*	*12 (30.8%)*	*0%*	*5 (25%)*	*-7.1%*
	6m	*14 (24.6%)*	*-6.7%*	*11 (28.9%)*	*-1.8%*	*3 (15.8%)*	*-16.4%*
Step 1 *(non-opioid + optional adjuvants)*	***v0***	*13 (16.3%)*		*8 (15.4%)*		*5 (17.9%)*	
	v15	*10 (15.9%)*	*-0.4%*	*5 (12.2%)*	*-3.2%*	*5 (22.7%)*	*4.9%*
	1m	*10 (15.6%)*	*-0.6%*	*5 (11.9%)*	*-3.5%*	*5 (22.7%)*	*4.9%*
	3m	*9 (15.3%)*	*-1%*	*4 (10.3%)*	*-5.1%*	*5 (25%)*	*7.1%*
	6m	*10 (17.5%)*	*1.3%*	*5 (13.2%)*	*-2.2%*	*5 (26.3%)*	*8.5%*
Step 2 *(weak opioid + non-opioid + optional adjuvants)*	***v0***	*6 (7.5%)*		*5 (9.6%)*		*1 (3.6%)*	
	v15	*6 (9.5%)*	*2%*	*5 (12.2%)*	*2.6%*	*1 (4.5%)*	*1%*
	1m	*6 (9.4%)*	*1.9%*	*5 (11.9%)*	*2.3%*	*1 (4.5%)*	*1%*
	3m	*4 (6.8%)*	*-0.7%*	*3 (7.7%)*	*-1.9%*	*1 (5%)*	*1.4%*
	6m	*3 (5.3%)*	*-2.2%*	*2 (5.3%)*	*-4.4%*	*1 (5.3%)*	*1.7%*
Step 3 *(strong opioid + non-opioid + optional adjuvants)*	***v0***	*14 (17.5%)*		*7 (13.5%)*		*7 (25%)*	
	v15	*10 (15.9%)*	*-1.6%*	*5 (12.2%)*	*-1.3%*	*5 (22.7%)*	*-2.3%*
	1m	*12 (18.8%)*	*1.3%*	*7 (16.7%)*	*3.2%*	*5 (22.7%)*	*-2.3%*

	3m	*12 (20.3%)*	*2.8%*	*8 (20.5%)*	*7.1%*	*4 (20%)*	*-5%*
	6m	*12 (21.1%)*	*3.6%*	*8 (21.1%)*	*7.6%*	*4 (21.1%)*	*-3.9%*

Medication usage over the course of the study for the FAS population. The table reports the number of participants for each level of medication consumption. Percentages are calculated with respect to the total number of participants still enrolled in the study. Percentage changes with respect to baseline percentages are also shown. Abbreviations: FAS, Full Analysis Set; PME, Phantom Motor Execution; PMI, Phantom Motor Imagery

Table S9: Descriptive summary of changes in medication consumption.

	Descriptive summary of medication use changes	
	**PME (n=52)**	**PMI (n=28)**
**Worsening of drug usage**		
V15	1 patient introduced acetaminophen (remains Step 3)	1 patient increase strong opioid (remains step 3)
	1 patient introduced strong opioid (from Step 1 to Step 3)	1 patient introduced NSAIDs (becomes Step 1 from only adjuvants)
1m	1 patient introduced adjuvant (remains step 1)	no change
3M	1 patient introduced strong opioids (from step 2 to step 3)	no change
6M	1 patient introduce NSAIDs (stays on step 2)	1 patient introduced adjuvant (remains on step 3)
	1 patient doubled the use of adjuvants and non-opioids (remains step 1)	
**Improvement of drug usage**		
V15	1 patient reduced weak opioid (remains Step 2)	1 patient decreased acetaminophen (remains step 1)
	1 patient reduced adjuvants and weak opioids (remains Step 2)	1 patient decreased adjuvants (medication free)
	1 patient quit adjuvants and reduced strong opioids (remains Step 3)	
	1 patient reduced one adjuvant (still on other adjuvants)	
	1 patient reduced acetaminophen (remains step 1)	
	1 patient quit adjuvant (remains Step 1 with other adjuvant unchanged)	
	1 patient decreased adjuvants	
	1 patient quit adjuvants (medication free)	
1m	1 patient quit one adjuvant (still on other adjuvants)	no change
	1 patient decreased adjuvants	
3M	1 patient decreased adjuvants	no change
6M	2 patients reduced adjuvants (remain on adjuvants)	1 patient quit adjuvants (medication free)

Abbreviations: PME, Phantom Motor Execution; PMI, Phantom Motor Imagery

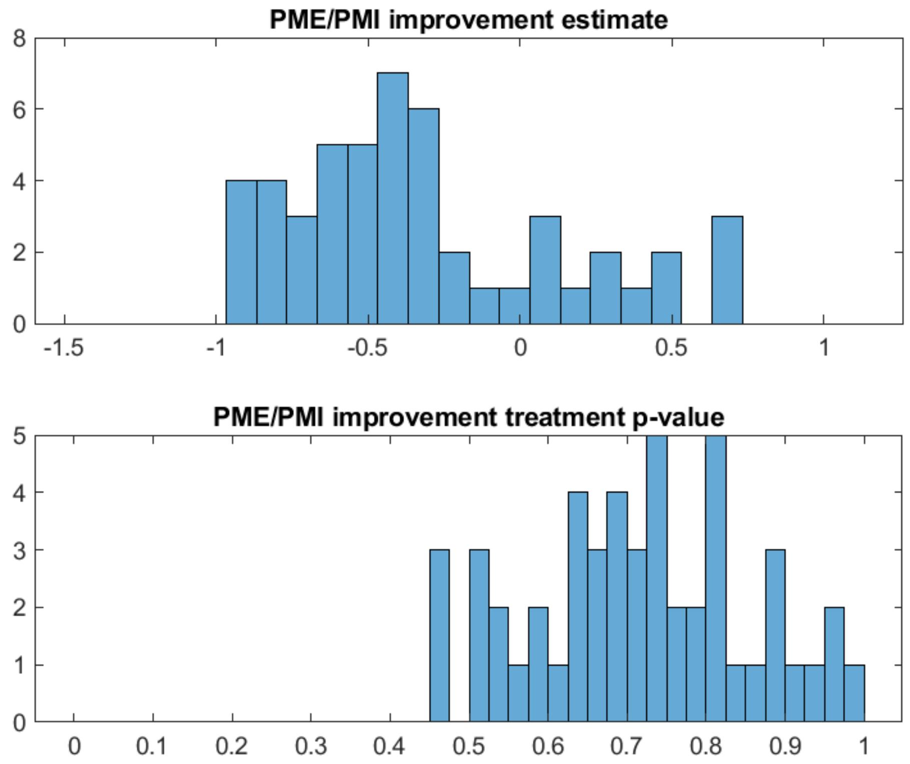

Figure S5: ANCOVA – Distribution of estimates and p-values

The ANCOVA analysis was carried out on the ITT population. The upper pane shows the distribution of the fifty linear model estimates (one for each imputed dataset) of the between-group differences (PME-PMI) for PRI scores. Negative estimates indicate larger pain reductions between baseline and end of treatment for the PMI group. The test resulted in 50 p-values, whose distribution is shown in the bottom pane. Abbreviations: PRI, Pain Rating Index; PME, Phantom Motor Execution; PMI, Phantom Motor Imagery; ITT, Intent-to-treat.

Table S10: Exploratory analyses.

	ITT population									
	PME				PMI				PME vs PMI	
	Mean (SD)	Change (%)	Mean SD (SD)	Treatment effect	Mean (SD)	Change (%)	Mean SD (SD)	Treatment effect	Mean (SD)	Treatment effect
**EuroQoL[2]**										
Change (v0 - v15)	-0.09±0.02	-15.9%	0.25±0.02	-0.36±0.06	-0.08±0.03	-15.0%	0.31±0.03	-0.26±0.08	-0.01±0.03	-0.03±0.12
Change (v0 - 1m)	-0.04±0.02	-6.9%	0.31±0.02	-0.13±0.07	-0.04±0.03	-7.5%	0.39±0.02	-0.10±0.08	0.00±0.04	0.00±0.10
Change (v0 - 3m)	-0.02±0.02	-2.7%	0.29±0.02	-0.05±0.06	-0.06±0.04	-11.9%	0.40±0.03	-0.16±0.09	0.05±0.04	0.15±0.12
Change (v0 - 6m)	-0.12±0.02	-21.7%	0.31±0.02	-0.39±0.06	-0.02±0.04	-3.9%	0.40±0.04	-0.05±0.10	-0.10±0.05	-0.29±0.14
**PCS**										
Change (v0 - v15)	2.14±0.29	20.9%	5.16±0.37	0.42±0.07	3.45±0.68	28.6%	6.94±0.67	0.50±0.12	-1.31±0.75	-0.23±0.13
Change (v0 - 1m)	1.75±0.39	17.1%	6.42±0.37	0.27±0.06	3.03±0.61	25.1%	6.21±0.57	0.49±0.12	-1.28±0.71	-0.20±0.11
Change (v0 - 3m)	3.38±0.35	33.0%	6.48±0.34	0.52±0.07	2.69±0.68	22.3%	7.01±0.66	0.39±0.12	0.70±0.72	0.10±0.11
Change (v0 - 6m)	2.80±0.35	27.3%	6.59±0.41	0.43±0.07	2.69±0.77	22.3%	6.27±0.90	0.44±0.16	0.11±0.85	0.02±0.13
**PDI**										

[2] Improvements in this variable are marked by increase in score

Change (v0 - v15)	9.15±01.15	29.1%	16.93±0.84	0.54±0.08	7.30±01.76	26.9%	19.57±01.21	0.38±0.10	1.85±02.00	0.10±0.11
Change (v0 - 1m)	8.55±01.10	27.2%	17.92±0.81	0.48±0.07	9.90±01.19	36.5%	13.44±0.97	0.74±0.11	-1.35±01.50	-0.08±0.09
Change (v0 - 3m)	7.69±01.17	24.5%	20.43±0.99	0.38±0.06	8.83±01.21	32.5%	12.38±0.99	0.72±0.11	-1.14±01.77	-0.06±0.10
Change (v0 - 6m)	7.99±0.97	25.4%	19.20±0.75	0.42±0.05	8.12±01.33	29.9%	12.73±01.50	0.65±0.12	-0.13±01.68	-0.01±0.10
PHQ										
Change (v0 - v15)	0.44±0.09	26.1%	1.63±0.09	0.27±0.06	0.73±0.13	38.3%	1.49±0.15	0.49±0.10	-0.28±0.17	-0.18±0.11
Change (v0 - 1m)	0.07±0.13	04.2%	1.67±0.09	0.04±0.08	0.03±0.16	01.6%	1.72±0.15	0.02±0.09	0.04±0.22	0.03±0.13
Change (v0 - 3m)	0.11±0.08	06.6%	1.66±0.07	0.07±0.05	0.18±0.13	09.2%	1.53±0.13	0.11±0.08	-0.06±0.16	-0.04±0.10
Change (v0 - 6m)	0.39±0.07	22.6%	1.23±0.06	0.31±0.06	-0.25±0.13	-13.0%	1.82±0.15	-0.14±0.07	0.63±0.15	0.43±0.10
PSEQ[2]										
Change (v0 - v15)	-0.65±0.15	-8.2%	3.21±0.18	-0.20±0.05	-1.36±0.22	-19.2%	2.99±0.20	-0.46±0.07	0.71±0.26	0.23±0.08
Change (v0 - 1m)	-0.64±0.16	-8.0%	3.77±0.15	-0.17±0.04	-0.64±0.30	-9.0%	3.10±0.27	-0.21±0.10	0.00±0.35	0.00±0.10
Change (v0 - 3m)	-0.25±0.18	-3.1%	3.23±0.19	-0.08±0.06	-1.11±0.23	-15.6%	2.00±0.23	-0.56±0.10	0.86±0.30	0.30±0.10

Change (v0 - 6m)	-0.88±0.17	-11.1%	3.25±0.19	-0.27±0.05	-0.71±0.30	-10.0%	2.89±0.27	-0.25±0.10	-0.17±0.34	-0.05±0.11
HCCQ										
Change (v0 - v15)	-1.44±0.47	-3.8%	7.65±0.75	-0.19±0.05	-0.30±0.52	-0.8%	5.21±0.53	-0.06±0.10	-1.14±0.70	-0.16±0.10
PPI										
Change (v0 - v15)	0.71±0.06	35.0%	1.29±0.05	0.55±0.06	0.62±0.09	33.3%	1.55±0.11	0.40±0.07	0.09±0.10	0.06±0.07
Change (v0 - 1m)	0.32±0.05	15.7%	1.46±0.07	0.22±0.04	0.04±0.12	01.9%	1.59±0.10	0.02±0.07	0.28±0.10	0.19±0.07
Change (v0 - 3m)	0.36±0.06	17.9%	1.48±0.06	0.25±0.04	-0.12±0.14	-6.2%	1.96±0.14	-0.06±0.07	0.48±0.14	0.29±0.09
Change (v0 - 6m)	0.13±0.05	06.2%	1.52±0.06	0.08±0.04	0.06±0.15	03.2%	1.73±0.13	0.04±0.09	0.07±0.17	0.04±0.10
NRS residual limb pain										
Change (v0 - v15)	0.71±0.05	49.3%	2.14±0.06	0.33±0.03	0.13±0.15	10.3%	2.58±0.21	0.05±0.05	0.58±0.15	0.25±0.05
Change (v0 - 1m)	0.36±0.08	24.9%	2.35±0.10	0.15±0.04	-0.01±0.13	-0.5%	2.30±0.17	0.00±0.05	0.37±0.15	0.16±0.06
Change (v0 - 3m)	-0.15±0.16	-10.2%	3.18±0.17	-0.04±0.05	-0.73±0.22	-58.3%	2.52±0.21	-0.28±0.07	0.58±0.29	0.20±0.10
Change (v0 - 6m)	-0.47±0.16	-32.7%	3.10±0.17	-0.15±0.05	-1.06±0.29	-84.6%	3.16±0.31	-0.33±0.06	0.59±0.30	0.19±0.09
NRS interference work										
Change (v0 - v15)	1.55±0.11	42.3%	3.80±0.14	0.41±0.04	1.63±0.18	50.3%	3.41±0.21	0.48±0.07	-0.08±0.21	-0.02±0.06
Change (v0 - 1m)	1.02±0.15	27.9%	3.96±0.17	0.26±0.04	0.38±0.28	11.8%	3.71±0.23	0.11±0.08	0.64±0.32	0.17±0.08

Change (v0 - 3m)	*0.31±0.18*	*08.4%*	*3.65±0.18*	*0.08±0.05*	*0.43±0.21*	*13.1%*	*2.98±0.20*	*0.14±0.07*	*-0.12±0.31*	*-0.03±0.09*
Change (v0 - 6m)	*0.72±0.15*	*19.7%*	*4.07±0.14*	*0.18±0.04*	*0.13±0.25*	*04.1%*	*3.12±0.26*	*0.05±0.08*	*0.59±0.30*	*0.16±0.08*
NRS interference ADL										
Change (v0 - v15)	*1.59±0.14*	*40.0%*	*3.91±0.13*	*0.41±0.04*	*2.72±0.15*	*59.0%*	*3.20±0.13*	*0.85±0.07*	*-1.12±0.21*	*-0.31±0.06*
Change (v0 - 1m)	*1.40±0.13*	*35.3%*	*4.17±0.11*	*0.34±0.03*	*1.46±0.24*	*31.6%*	*3.76±0.21*	*0.39±0.07*	*-0.05±0.28*	*-0.01±0.07*
Change (v0 - 3m)	*-0.04±0.20*	*-1.0%*	*4.21±0.14*	*-0.01±0.05*	*1.38±0.17*	*30.0%*	*2.79±0.21*	*0.50±0.08*	*-1.42±0.28*	*-0.38±0.08*
Change (v0 - 6m)	*0.34±0.17*	*08.5%*	*4.06±0.15*	*0.08±0.04*	*1.67±0.26*	*36.2%*	*3.39±0.21*	*0.49±0.09*	*-1.33±0.30*	*-0.35±0.08*
NRS interference sleep										
Change (v0 - v15)	*1.89±0.11*	*45.8%*	*4.18±0.13*	*0.45±0.03*	*3.85±0.19*	*58.2%*	*2.79±0.10*	*1.38±0.09*	*-1.96±0.24*	*-0.52±0.06*
Change (v0 - 1m)	*1.13±0.13*	*27.4%*	*4.21±0.15*	*0.27±0.03*	*3.13±0.19*	*47.3%*	*3.23±0.12*	*0.97±0.08*	*-2.00±0.23*	*-0.51±0.06*
Change (v0 - 3m)	*0.48±0.16*	*11.6%*	*4.01±0.17*	*0.12±0.04*	*2.32±0.23*	*35.1%*	*3.25±0.23*	*0.72±0.10*	*-1.84±0.28*	*-0.49±0.07*
Change (v0 - 6m)	*0.39±0.14*	*09.5%*	*3.98±0.17*	*0.10±0.04*	*1.91±0.23*	*29.0%*	*2.99±0.22*	*0.65±0.10*	*-1.52±0.27*	*-0.41±0.07*
EXPECT-SF mean[2]										

Change (v0 - v1)	-0.02±0.08	-0.3%	1.87±0.05	-0.01±0.05	0.23±0.16	03.9%	1.52±0.09	0.15±0.10	-0.25±0.17	-0.14±0.10
EXPECT-SF Q1[2]										
Visit 0	8.20±0.07	nan	1.73±0.05	nan	8.12±0.11	nan	1.87±0.09	nan	0.08±0.12	0.03±0.02
Visit 1	8.38±0.12	nan	1.73±0.06	nan	7.84±0.16	nan	1.81±0.11	nan	0.54±0.19	0.14±0.05
Change (v0 - v1)	-0.17±0.14	-2.1%	1.89±0.11	-0.09±0.08	0.28±0.18	03.4%	1.91±0.20	0.14±0.09	-0.45±0.22	-0.24±0.11
EXPECT-SF Q2[2]										
Visit 0	6.04±0.09	nan	2.23±0.06	nan	5.85±0.13	nan	2.41±0.07	nan	0.19±0.16	0.04±0.03
Visit 1	6.33±0.13	nan	1.99±0.09	nan	5.42±0.17	nan	2.24±0.11	nan	0.91±0.20	0.21±0.04
Change (v0 - v1)	-0.29±0.15	-4.8%	2.51±0.12	-0.12±0.06	0.43±0.21	07.3%	2.01±0.21	0.22±0.11	-0.72±0.24	-0.31±0.11
EXPECT-SF Q3[2]										
Visit 0	6.52±0.10	nan	1.95±0.09	nan	5.84±0.15	nan	2.90±0.08	nan	0.68±0.18	0.14±0.04
Visit 1	6.29±0.14	nan	2.38±0.07	nan	5.92±0.18	nan	2.36±0.12	nan	0.37±0.23	0.08±0.04
Change (v0 - v1)	0.23±0.16	03.5%	2.47±0.14	0.09±0.06	-0.07±0.25	-1.3%	2.34±0.22	-0.03±0.10	0.31±0.28	0.13±0.12
EXPECT-SF Q4[2]										
Visit 0	6.31±0.10	nan	2.34±0.07	nan	5.91±0.13	nan	2.60±0.09	nan	0.39±0.16	0.08±0.03
Visit 1	6.30±0.14	nan	2.35±0.08	nan	5.58±0.16	nan	1.97±0.12	nan	0.72±0.19	0.15±0.04

Change (v0 - v1)	*0.01±0.19*	*0.2%*	*2.54±0.15*	*0.00±0.07*	*0.34±0.22*	*05.7%*	*1.98±0.17*	*0.17±0.11*	*-0.33±0.27*	*-0.14±0.11*
OTA[2]										
Visit 1	*21.11±0.17*	*nan*	*4.72±0.31*	*nan*	*19.32±0.30*	*nan*	*5.34±0.38*	*nan*	*1.79±0.34*	*0.36±0.07*
PM NRS										
Change (v0 - v15)	*0.19±0.14*	*03.4%*	*3.66±0.13*	*0.05±0.04*	*0.15±0.14*	*02.3%*	*2.05±0.11*	*0.07±0.06*	*0.04±0.20*	*0.01±0.06*
Change (v0 - 1m)	*0.00±0.11*	*0.1%*	*3.46±0.11*	*0.00±0.03*	*0.20±0.11*	*03.0%*	*1.55±0.07*	*0.13±0.07*	*-0.20±0.17*	*-0.07±0.06*
Change (v0 - 3m)	*0.25±0.13*	*04.5%*	*3.60±0.12*	*0.07±0.03*	*0.07±0.22*	*01.1%*	*2.52±0.14*	*0.03±0.08*	*0.18±0.26*	*0.05±0.08*
Change (v0 - 6m)	*0.26±0.15*	*04.5%*	*3.46±0.11*	*0.07±0.04*	*0.09±0.15*	*01.3%*	*2.72±0.10*	*0.03±0.05*	*0.17±0.23*	*0.05±0.07*
PLS NRS										
Change (v0 - v15)	*0.53±0.15*	*10.7%*	*3.91±0.15*	*0.14±0.04*	*-1.49±0.20*	*-29.2%*	*3.47±0.21*	*-0.43±0.08*	*2.02±0.26*	*0.54±0.08*
Change (v0 - 1m)	*0.69±0.18*	*14.1%*	*3.96±0.15*	*0.18±0.05*	*-0.33±0.20*	*-6.4%*	*3.98±0.20*	*-0.08±0.05*	*1.02±0.30*	*0.26±0.08*
Change (v0 - 3m)	*1.09±0.18*	*22.2%*	*4.02±0.15*	*0.27±0.05*	*-0.96±0.25*	*-18.8%*	*3.77±0.20*	*-0.26±0.08*	*2.05±0.32*	*0.52±0.09*
Change (v0 - 6m)	*0.27±0.20*	*05.5%*	*3.80±0.14*	*0.07±0.05*	*-0.35±0.26*	*-6.9%*	*3.87±0.23*	*-0.09±0.07*	*0.62±0.31*	*0.16±0.08*

Summary of exploratory outcome variables analyzed on the ITT population. Abbreviations: ITT, Intent-to-treat; PME, Phantom Motor Execution; PMI, Phantom Motor Imagery; PP, Per Protocol; EQ-5D-5L, EuroQol-5D-5L; HCCQ, Health Care Climate Questionnaire; PCS, Pain Catastrophizing Scale; PDI, Pain Disability Index; PHQ, Patient Health Questionnaire; PSEQ, Pain Self-Efficacy Questionnaire; PPO, Present Pain Intensity; NRS, Numeric Rating Scale; EXPECT, Expectations for

Complementary and Alternative Medicine Treatments; NRS, Numeric Rating Scale; OAT, Opinion About Treatment; PM, Phantom Movement ability; PLS, Phantom Limb Sensations; ADL, Activities of the Daily Living; Q#, Question

Table S11: Analysis of dichotomous secondary and exploratory variables.

		PME		PMI		
		**# patients**	**% change**	**# patients**	**% change**	**Odds ratio**
**PRI Significant improvement (50%)**						
	Change (v0 - v15) in ITT	37.42±0.97 (52)	72%	19.48±0.79 (28)	70%	1.14±0.19
	Change (v0 - v15) in PP	27(38)	71%	11(17)	65%	1.34
**Telescoping improvement (lengthening)**						
	Change (v0 - v15)	12.40±0.90 (24)	52%	4.86±0.76 (17)	29%	2.69±0.64
	Change (v0 - 1m)	13.14±0.95 (24)	55%	6.32±0.91 (17)	37%	2.06±0.58
	Change (v0 - 3m)	14.42±0.61 (24)	60%	8.16±0.77 (17)	48%	1.59±0.30
	Change (v0 - 6m)	11.50±0.74 (24)	48%	8.60±0.95 (17)	51%	0.88±0.20
**Telescoping worsening (shrinking)**						
	Change (v0 - v15)	9.60±1.09 (52)	19%	4.72±0.83 (28)	17%	1.16±0.28
	Change (v0 - 1m)	12.24±1.17 (52)	24%	3.64±0.69 (28)	13%	2.14±0.48
	Change (v0 - 3m)	12.98±1.10 (52)	25%	3.24±1.04 (28)	12%	2.87±1.10
	Change (v0 - 6m)	14.80±1.36 (52)	29%	6.30±1.34 (28)	23%	1.47±0.51
**Telescoping stable**						
	Change (v0 - v15)	30.±1.25 (52)	58%	18.42±0.93 (28)	66%	0.72±0.12
	Change (v0 - 1m)	26.62±1.41 (52)	51%	18.04±0.97 (28)	64%	0.59±0.10
	Change (v0 - 3m)	24.60±1.23 (52)	47%	16.60±1.12 (28)	59%	0.63±0.12
	Change (v0 - 6m)	25.70±1.31 (52)	49%	13.10±1.33 (28)	47%	1.14±0.24
**PLP frequency improvement**						
	Change (v0 - v15)	15.08±1.64 (52)	29%	7.96±1.12 (28)	28%	1.06±0.25
	Change (v0 - 1m)	27.86±0.93 (52)	54%	10.12±1(28)	36%	2.08±0.36
	Change (v0 - 3m)	24.16±1.30 (52)	47%	12.08±0.80 (28)	43%	1.16±0.16
	Change (v0 - 6m)	26.54±1.68 (52)	51%	8.58±1.30 (28)	31%	2.47±0.69
**PLP frequency worsening**						
	Change (v0 - v15)	3.10±0.91 (52)	6%	3.90±1.02 (28)	14%	0.43±0.18
	Change (v0 - 1m)	4.92±0.88 (52)	10%	4.12±0.72 (28)	15%	0.63±0.16
	Change (v0 - 3m)	2.60±0.88 (52)	5%	3.06±0.55 (28)	11%	0.44±0.17
	Change (v0 - 6m)	3.02±0.98 (52)	6%	4.76±0.87 (28)	17%	0.31±0.13
**PLP frequency stable**						
	Change (v0 - v15)	33.82±1.57 (52)	65%	16.14±1.29 (28)	58%	1.41±0.36
	Change (v0 - 1m)	19.22±1.15 (52)	37%	13.76±0.98 (28)	49%	0.62±0.10
	Change (v0 - 3m)	25.24±1.30 (52)	49%	12.86±0.88 (28)	46%	1.12±0.17
	Change (v0 - 6m)	22.44±1.49 (52)	43%	14.66±1.24 (28)	52%	0.71±0.15

Significant improvement in PRI is interpreted as the presence of a clinically meaningful pain reduction. Telescoping is interpreted as the presence of improvement (perceived lengthening of the phantom limb), worsening (shortening) and

stability (no change in perceived length). Frequency of PLP is interpreted as the presence of improvement (reduced frequency of PLP episodes), worsening (increased frequency) or stability (no change). At end of treatment, patients in the PME group were more likely to report improvement in telescoping (odds ratio 2.69±0.64) however the effect reversed by the end of the follow up period (odds ratio 0.88±0.20). Interestingly, patient in the PME group were also more likely to report worsening of telescoping (odds ratio 1.19±0.34) and this effect was enhanced by the end of the follow up period (odds ratio 1.87±0.58). This suggest that overall, there was more variability in the PME group. Also worth noticing that PME participants were less likely to have worsened pain frequency (odds ratio 0.43±0.18).

Abbreviations: ITT, Intent-to-treat; PP, Per Protocol; PME, Phantom Motor Execution; PMI, Phantom Motor Imagery; PRI, Pain Rating Index; Sig PRI, Clinically Significant reduction in PRI

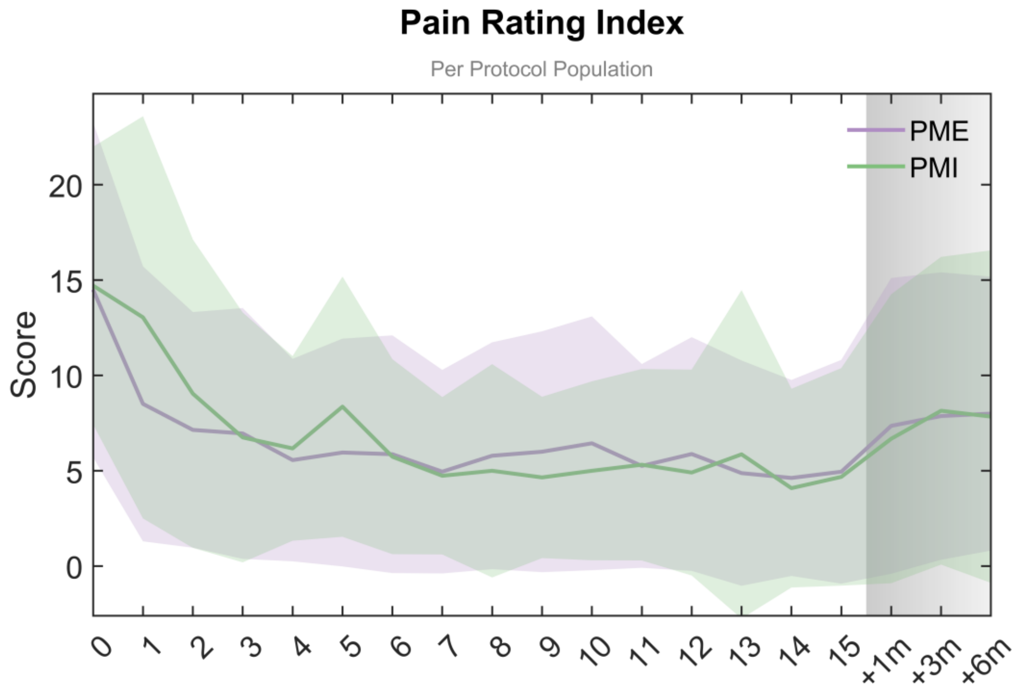

Figure S6: PRI trend (PP)

The graph represents the trend over the study of mean and standard deviation of PRI scores for PME and PMI. The data is taken from the PP population and the plot is intended to be compared to Figure 3 in the manuscript where the same data is shown for the ITT population. This allows the reader to qualitatively assess the impact of the stochastic imputation on the fidelity of the distribution of the data and whether it distorts it. Visit 0: baseline, Visit 1-15: treatment session, 1m, +3m, +6m: 1-,3- and 6-month post-treatment assessment. Abbreviations: PP, Per Protocol;

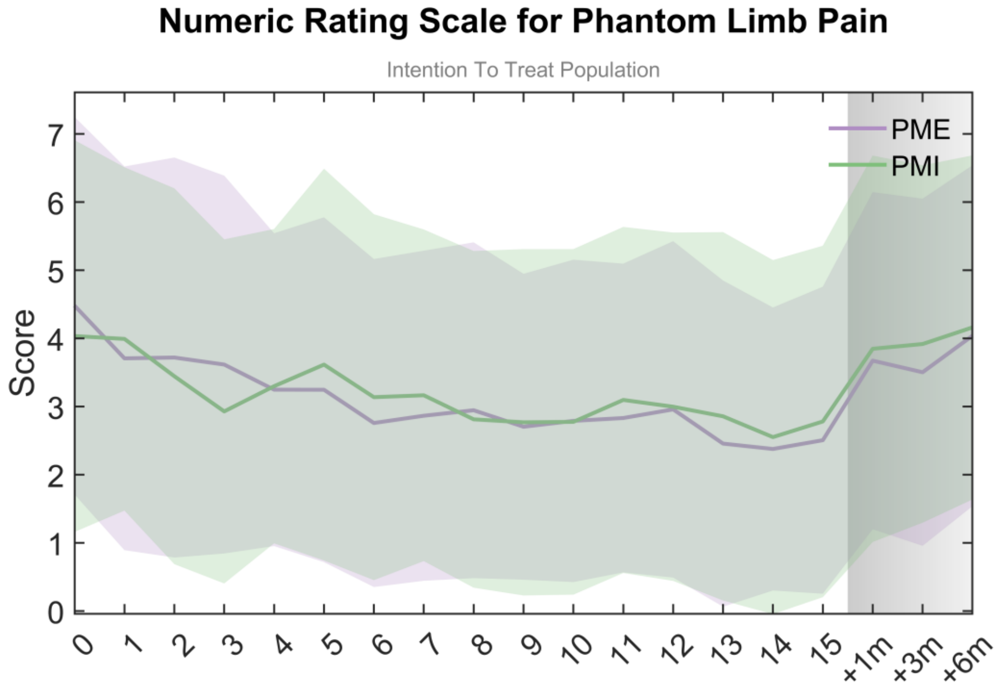

Figure S7: NRS PLP trend (ITT)

The trend of mean and standard deviation of NRS scores [0-10] for PLP for PME and PMI throughout the study. Visit 0: baseline, Visit 1-15: treatment session, 1m, +3m, +6m: 1-,3- and 6-month post-treatment assessment. Abbreviations: ITT, Intent-to-treat; PME, Phantom Motor Execution; PMI, Phantom Motor Imagery; NRS, Numeric Rating Scale

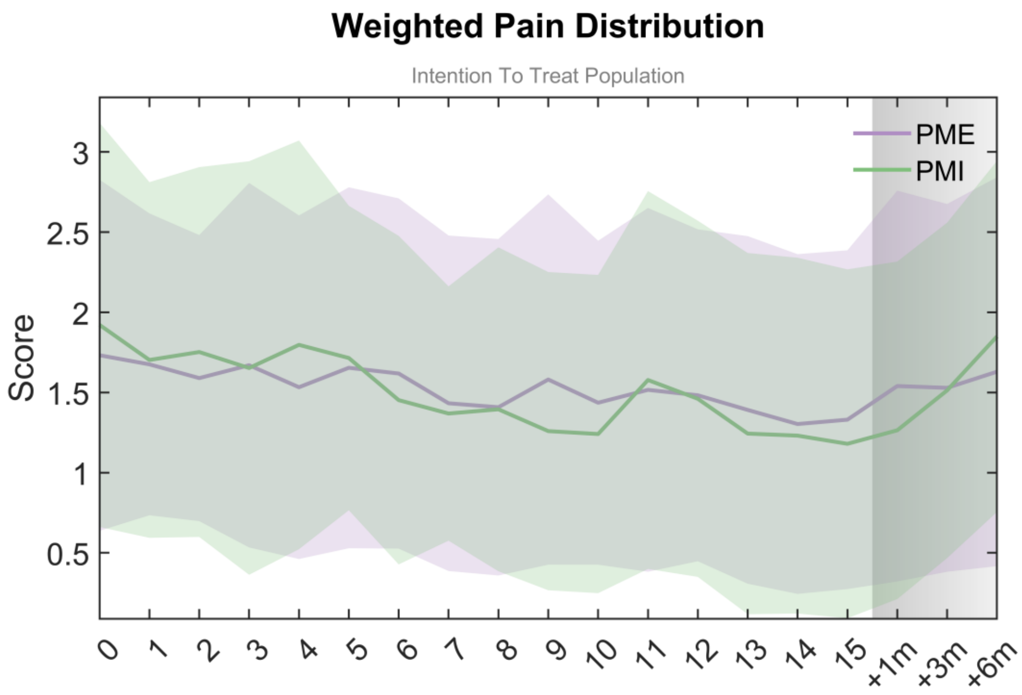

Figure S8: WPD trend (ITT)

The trend of mean and standard deviation of WPD scores for PME and PMI throughout the study. Visit 0: baseline, Visit 1-15: treatment session, 1m, +3m, +6m: 1-,3- and 6-month post-treatment assessment. Abbreviations: ITT, Intent-to-treat; PME, Phantom Motor Execution; PMI, Phantom Motor Imagery; WPD, Weighted Pain Distribution

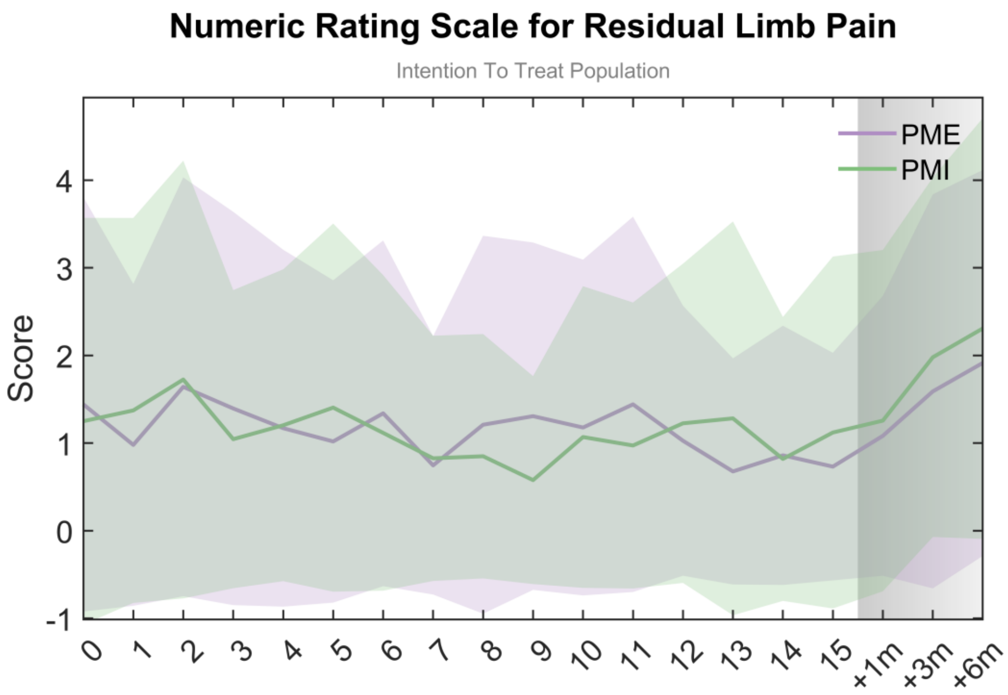

Figure S9: NRS Residual Limb Pain trend (ITT)

The trend of mean and standard deviation of NRS scores [0-10] of residual limb pain for PME and PMI throughout the study. Visit 0: baseline, Visit 1-15: treatment session, 1m, +3m, +6m: 1-,3- and 6-month post-treatment assessment. Abbreviations: ITT, Intent-to-treat; PME, Phantom Motor Execution; PMI, Phantom Motor Imagery; NRS, Numeric Rating Scale

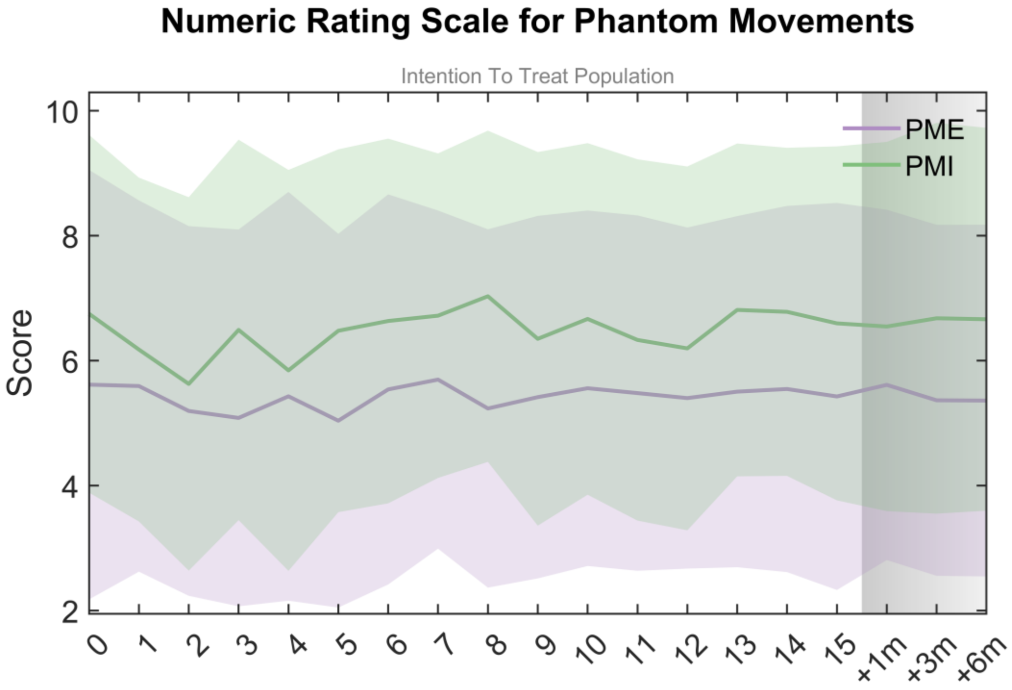

Figure S10: NRS PM (ITT)

The trend of the mean and standard deviation of NRS scores [0-10] rating the ability to move the phantom limb under voluntary control. A self-rated score of 10 indicates a perceived full control over the movements of the phantom limb. Scores are shown for PME and PMI throughout the study. Visit 0: Baseline, Visit 1-15: treatment session, 1m, +3m, +6m: 1-,3- and 6-month post-treatment assessment. Abbreviations: ITT, Intent-to-treat; PME, Phantom Motor Execution; PMI, Phantom Motor Imagery; NRS, Numeric Rating Scale; PM, Phantom Motor ability

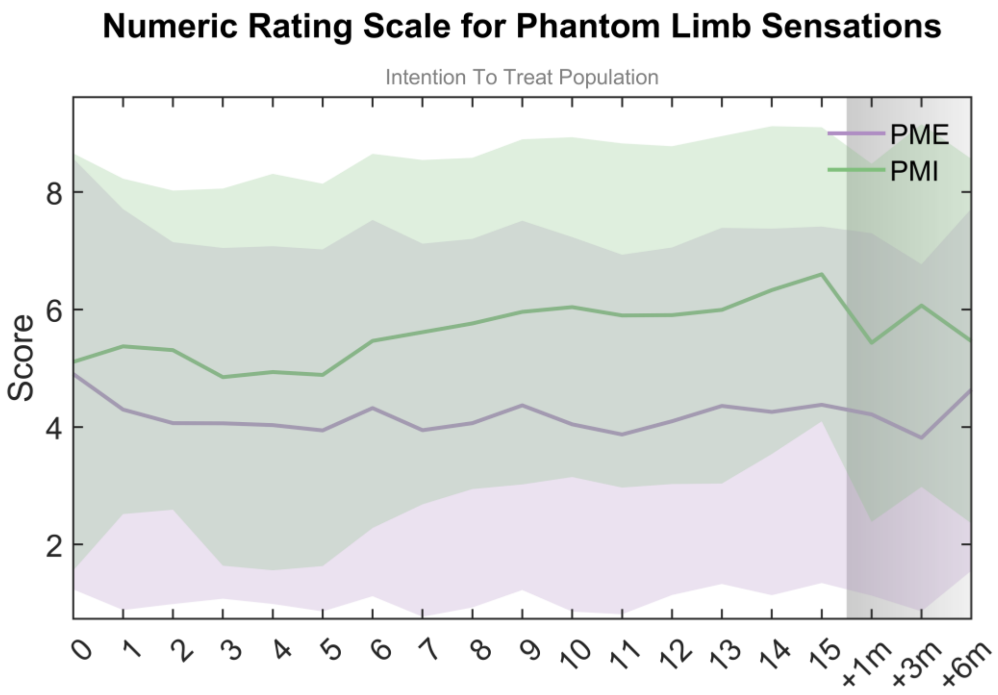

Figure S11: NRS PLS trend (ITT)

The trend of mean and standard deviation of NRS scores of phantom limb sensations for PME and PMI throughout the study. Visit 0: baseline, Visi t 1-15: treatment session, 1m, +3m, +6m: 1-,3- and 6-month post-treatment assessment. Abbreviations: ITT, Intent-to-treat; PME, Phantom Motor Execution; PMI, Phantom Motor Imagery; NRS, Numeric Rating Scale; PLS, Phantom Limb Sensations

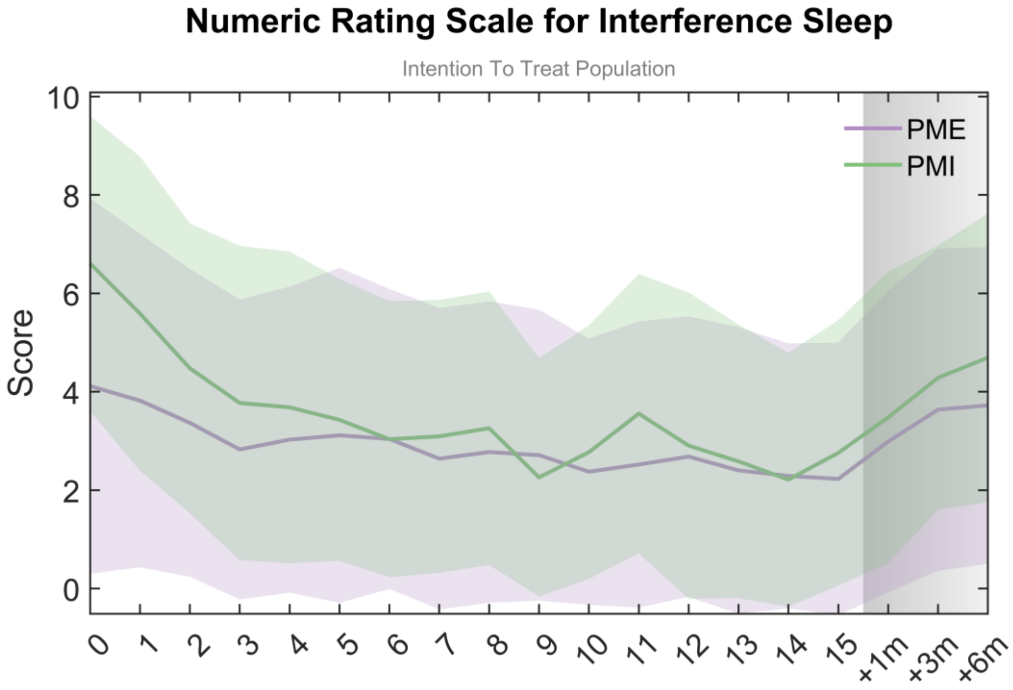

Figure S12: NRS Interference with sleep (ITT)

The trend of mean and standard deviation of NRS scores [0-10] for pain interference with sleep for PME and PMI throughout the study. Visit 0: baseline, Visit 1-15: treatment session, 1m, +3m, +6m: 1-,3- and 6-month post-treatment assessment. Abbreviations: ITT, Intent-to-treat; PME, Phantom Motor Execution; PMI, Phantom Motor Imagery; NRS, Numeric Rating Scale

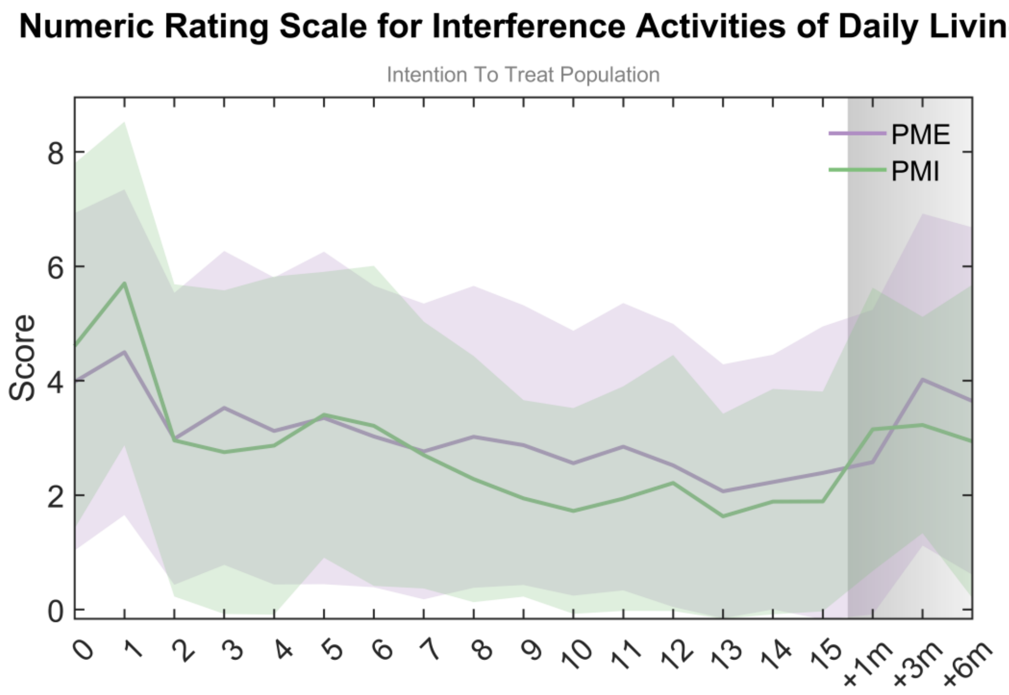

Figure S13: NRS Interference with activities of daily living (ITT)

The trend of mean and standard deviation of NRS scores [0-10] for pain interference with activities of the daily living for PME and PMI throughout the study. Visit 0: baseline, Visit 1-15: treatment session, Visit 16-18, 1m, +3m, +6m: 1-,3- and 6-month post-treatment assessment. Abbreviations: ITT, Intent-to-treat; PME, Phantom Motor Execution; PMI, Phantom Motor Imagery; NRS, Numeric Rating Scale; ADL, Activities of the Daily Living

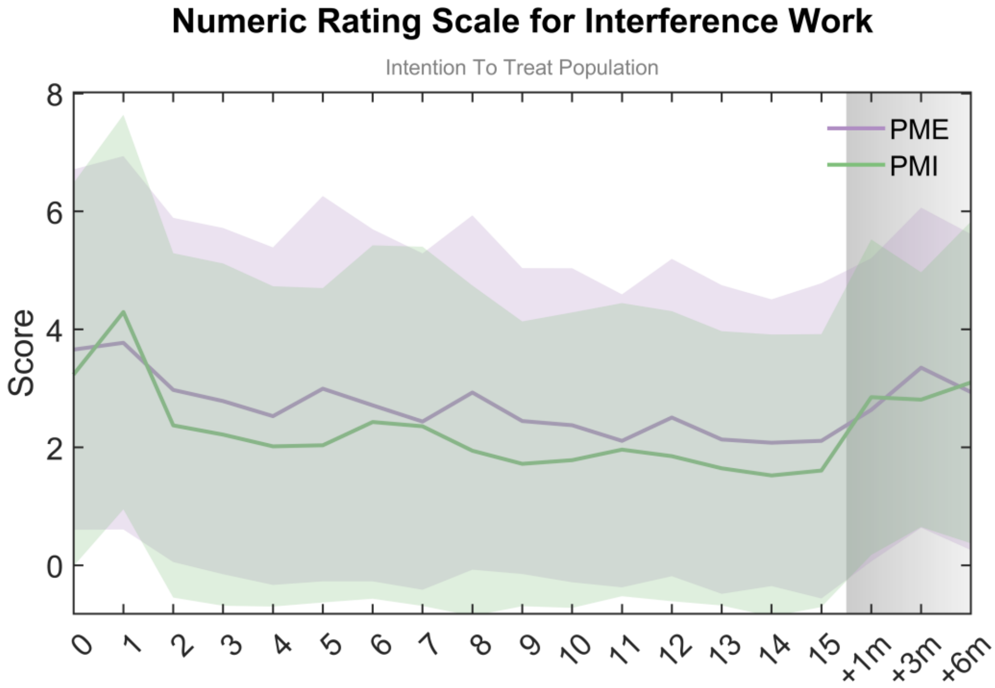

Figure S14: NRS Interference with work (ITT)

The trend of mean and standard deviation of NRS scores [0-10] for pain interference with working life for PME and PMI throughout the study. Visit 0: baseline, Visit 1-15: treatment session, +1m, +3m, +6m: 1-,3- and 6-month post-treatment assessment. Abbreviations: ITT, Intent-to-treat; PME, Phantom Motor Execution; PMI, Phantom Motor Imagery; NRS, Numeric Rating Scale

Table S12: McGill pain descriptors throughout the study in the FAS population

	**Visit 0**			**Visit 15**			**1M**			**3M**			**6M**		
Sum	*Overall*	*PME*	*PMI*	*Overall*	*PME*	*PMI*	*Overall*	*PME*	*PMI*	*Overall*	*PME*	*PMI*	*Overall*	*PME*	*PMI*
Throbbing	*36*	*23*	*13*	*14*	*10*	*4*	*24*	*16*	*8*	*22*	*16*	*6*	*22*	*17*	*5*
	(45%)	*(44.2%)*	*(46.4%)*	*(22.2%)*	*(24.4%)*	*(18.2%)*	*(37.5%)*	*(38.1%)*	*(36.4%)*	*(37.3%)*	*(41%)*	*(30%)*	*(39.3%)*	*(45.9%)*	*(26.3%)*
Shooting	*38*	*23*	*15*	*9*	*6*	*3*	*14*	*11*	*3*	*14*	*10*	*4*	*18*	*13*	*5*
	(47.5%)	*(44.2%)*	*(53.6%)*	*(14.3%)*	*(14.6%)*	*(13.6%)*	*(21.9%)*	*(26.2%)*	*(13.6%)*	*(23.7%)*	*(25.6%)*	*(20%)*	*(32.1%)*	*(35.1%)*	*(26.3%)*
Stabbing	*48*	*31*	*17*	*23*	*15*	*8*	*24*	*17*	*7*	*26*	*18*	*8*	*27*	*20*	*7*
	(60%)	*(59.6%)*	*(60.7%)*	*(36.5%)*	*(36.6%)*	*(36.4%)*	*(37.5%)*	*(40.5%)*	*(31.8%)*	*(44.1%)*	*(46.2%)*	*(40%)*	*(48.2%)*	*(54.1%)*	*(36.8%)*
Cramping	*50*	*33*	*17*	*23*	*15*	*8*	*21*	*14*	*7*	*28*	*18*	*10*	*22*	*15*	*7*
	(62.5%)	*(63.5%)*	*(60.7%)*	*(36.5%)*	*(36.6%)*	*(36.4%)*	*(32.8%)*	*(33.3%)*	*(31.8%)*	*(47.5%)*	*(46.2%)*	*(50%)*	*(39.3%)*	*(40.5%)*	*(36.8%)*
Sharp	*40*	*26*	*14*	*15*	*10*	*5*	*19*	*15*	*4*	*22*	*16*	*6*	*14*	*8*	*6*
	(50%)	*(50%)*	*(50%)*	*(23.8%)*	*(24.4%)*	*(22.7%)*	*(29.7%)*	*(35.7%)*	*(18.2%)*	*(37.3%)*	*(41%)*	*(30%)*	*(25%)*	*(21.6%)*	*(31.6%)*
Gnawing	*29*	*22*	*7*	*9*	*8*	*1*	*17*	*12*	*5*	*17*	*14*	*3*	*16*	*11*	*5*
	(36.3%)	*(42.3%)*	*(25%)*	*(14.3%)*	*(19.5%)*	*(4.5%)*	*(26.6%)*	*(28.6%)*	*(22.7%)*	*(28.8%)*	*(35.9%)*	*(15%)*	*(28.6%)*	*(29.7%)*	*(26.3%)*
Hot-Burning	*38*	*25*	*13*	*21*	*12*	*9*	*23*	*13*	*10*	*25*	*14*	*11*	*17*	*14*	*3*
	(47.5%)	*(48.1%)*	*(46.4%)*	*(33.3%)*	*(29.3%)*	*(40.9%)*	*(35.9%)*	*(31%)*	*(45.5%)*	*(42.4%)*	*(35.9%)*	*(55%)*	*(30.4%)*	*(37.8%)*	*(15.8%)*
Aching	*56*	*37*	*19*	*22*	*16*	*6*	*31*	*21*	*10*	*28*	*21*	*7*	*31*	*21*	*10*
	(70%)	*(71.2%)*	*(67.9%)*	*(34.9%)*	*(39%)*	*(27.3%)*	*(48.4%)*	*(50%)*	*(45.5%)*	*(47.5%)*	*(53.8%)*	*(35%)*	*(55.4%)*	*(56.8%)*	*(52.6%)*
Heavy	*42*	*25*	*17*	*15*	*10*	*5*	*17*	*13*	*4*	*21*	*13*	*8*	*17*	*11*	*6*
	(52.5%)	*(48.1%)*	*(60.7%)*	*(23.8%)*	*(24.4%)*	*(22.7%)*	*(26.6%)*	*(31%)*	*(18.2%)*	*(35.6%)*	*(33.3%)*	*(40%)*	*(30.4%)*	*(29.7%)*	*(31.6%)*
	29	*19*	*10*	*6*	*2*	*4*	*15*	*9*	*6*	*17*	*11*	*6*	*22*	*16*	*6*

Tender	*(36.3%)*	*(36.5%)*	*(35.7%)*	*(9.5%)*	*(4.9%)*	*(18.2%)*	*(23.4%)*	*(21.4%)*	*(27.3%)*	*(28.8%)*	*(28.2%)*	*(30%)*	*(39.3%)*	*(43.2%)*	*(31.6%)*
Splitting	*30*	*18*	*12*	*8*	*6*	*2*	*8*	*5*	*3*	*12*	*7*	*5*	*10*	*6*	*4*
	(37.5%)	*(34.6%)*	*(42.9%)*	*(12.7%)*	*(14.6%)*	*(9.1%)*	*(12.5%)*	*(11.9%)*	*(13.6%)*	*(20.3%)*	*(17.9%)*	*(25%)*	*(17.9%)*	*(16.2%)*	*(21.1%)*
Tiring-Exhausting	*57*	*35*	*22*	*18*	*13*	*5*	*27*	*19*	*8*	*25*	*17*	*8*	*25*	*18*	*7*
	(71.3%)	*(67.3%)*	*(78.6%)*	*(28.6%)*	*(31.7%)*	*(22.7%)*	*(42.2%)*	*(45.2%)*	*(36.4%)*	*(42.4%)*	*(43.6%)*	*(40%)*	*(44.6%)*	*(48.6%)*	*(36.8%)*
Sickening	*9*	*4*	*5*	*6*	*4*	*2*	*5*	*4*	*1*	*2*	*1*	*1*	*2*	*2*	*0*
	(11.3%)	*(7.7%)*	*(17.9%)*	*(9.5%)*	*(9.8%)*	*(9.1%)*	*(7.8%)*	*(9.5%)*	*(4.5%)*	*(3.4%)*	*(2.6%)*	*(5%)*	*(3.6%)*	*(5.4%)*	*(0%)*
Fearful	*22*	*17*	*5*	*6*	*4*	*2*	*11*	*9*	*2*	*8*	*6*	*2*	*7*	*6*	*1*
	(27.5%)	*(32.7%)*	*(17.9%)*	*(9.5%)*	*(9.8%)*	*(9.1%)*	*(17.2%)*	*(21.4%)*	*(9.1%)*	*(13.6%)*	*(15.4%)*	*(10%)*	*(12.5%)*	*(16.2%)*	*(5.3%)*
Punishing-Cruel	*27*	*19*	*8*	*4*	*2*	*2*	*8*	*6*	*2*	*7*	*4*	*3*	*7*	*3*	*4*
	(33.8%)	*(36.5%)*	*(28.6%)*	*(6.3%)*	*(4.9%)*	*(9.1%)*	*(12.5%)*	*(14.3%)*	*(9.1%)*	*(11.9%)*	*(10.3%)*	*(15%)*	*(12.5%)*	*(8.1%)*	*(21.1%)*

Number (and percent) of participants reporting each of the McGill pain descriptors at baseline, end of treatment and follow-up assessments. Abbreviations: FAS, Ful Analysis Set; PME, Phantom Motor Execution; PMI, Phantom Motor Imagery; NRS, Numeric Rating Scale

Table S13: Frequency of PLP in the FAS population

	**v0**			**v15**			**1M**			**3M**			**6M**		
	**Overall**	**PME**	**PMI**	**Overall**	**PME**	**PMI**	**Overall**	**PME**	**PMI**	**Overall**	**PME**	**PMI**	**Overall**	**PME**	**PMI**
**Constantly**	*43*	*29*	*14*	*30*	*21*	*9*	*28*	*18*	*10*	*25*	*17*	*8*	*28*	*17*	*11*
	(53.8%)	*(55.8%)*	*(50%)*	*(47.6%)*	*(51.2%)*	*(40.9%)*	*(43.8%)*	*(42.9%)*	*(45.5%)*	*(42.4%)*	*(43.6%)*	*(40%)*	*(50%)*	*(45.9%)*	*(57.9%)*
**few/day**	*13*	*8*	*5*	*15*	*8*	*7*	*19*	*14*	*5*	*13*	*10*	*3*	*11*	*8*	*3*
	(16.3%)	*(15.4%)*	*(17.9%)*	*(23.8%)*	*(19.5%)*	*(31.8%)*	*(29.7%)*	*(33.3%)*	*(22.7%)*	*(22%)*	*(25.6%)*	*(15%)*	*(19.6%)*	*(21.6%)*	*(15.8%)*
**once/day**	*6*	*5*	*1*	*4*	*3*	*1*	*2*	*1*	*1*	*4*	*2*	*2*	*3*	*3*	*0*
	(7.5%)	*(9.6%)*	*(3.6%)*	*(6.3%)*	*(7.3%)*	*(4.5%)*	*(3.1%)*	*(2.4%)*	*(4.5%)*	*(6.8%)*	*(5.1%)*	*(10%)*	*(5.4%)*	*(8.1%)*	*(0%)*
**few/week**	*11*	*5*	*6*	*8*	*4*	*4*	*3*	*1*	*2*	*8*	*5*	*3*	*6*	*3*	*3*
	(13.8%)	*(9.6%)*	*(21.4%)*	*(12.7%)*	*(9.8%)*	*(18.2%)*	*(4.7%)*	*(2.4%)*	*(9.1%)*	*(13.6%)*	*(12.8%)*	*(15%)*	*(10.7%)*	*(8.1%)*	*(15.8%)*
**once/week**	*5*	*3*	*2*	*2*	*2*	*0*	*4*	*3*	*1*	*1*	*0*	*1*	*1*	*1*	*0*
	(6.3%)	*(5.8%)*	*(7.1%)*	*(3.2%)*	*(4.9%)*	*(0%)*	*(6.3%)*	*(7.1%)*	*(4.5%)*	*(1.7%)*	*(0%)*	*(5%)*	*(1.8%)*	*(2.7%)*	*(0%)*
**few/month**	*1*	*1*	*0*	*1*	*1*	*0*	*2*	*2*	*0*	*3*	*2*	*1*	*5*	*4*	*1*
	(1.3%)	*(1.9%)*	*(0%)*	*(1.6%)*	*(2.4%)*	*(0%)*	*(3.1%)*	*(4.8%)*	*(0%)*	*(5.1%)*	*(5.1%)*	*(5%)*	*(8.9%)*	*(10.8%)*	*(5.3%)*
**once/month**	*1*	*1*	*0*	*2*	*1*	*1*	*3*	*2*	*1*	*4*	*3*	*1*	*0*	*0*	*0*
	(1.3%)	*(1.9%)*	*(0%)*	*(3.2%)*	*(2.4%)*	*(4.5%)*	*(4.7%)*	*(4.8%)*	*(4.5%)*	*(6.8%)*	*(7.7%)*	*(5%)*	*(0%)*	*(0%)*	*(0%)*
**Never**	*0*	*0*	*0*	*0*	*0*	*0*	*3*	*1*	*2*	*1*	*0*	*1*	*3*	*2*	*1*
	(0%)	*(0%)*	*(0%)*	*(0%)*	*(0%)*	*(0%)*	*(4.7%)*	*(2.4%)*	*(9.1%)*	*(1.7%)*	*(0%)*	*(5%)*	*(5.4%)*	*(5.4%)*	*(5.3%)*

Participants-reported frequency of PLP episodes at baseline, end of treatment and follow-up assessments Number of participants reporting each of the McGill pain descriptors at baseline, end of treatment and follow-up assessments. Abbreviations: FAS, Ful Analysis Set; PME, Phantom Motor Execution; PMI, Phantom Motor Imagery; NRS, Numeric Rating Scale

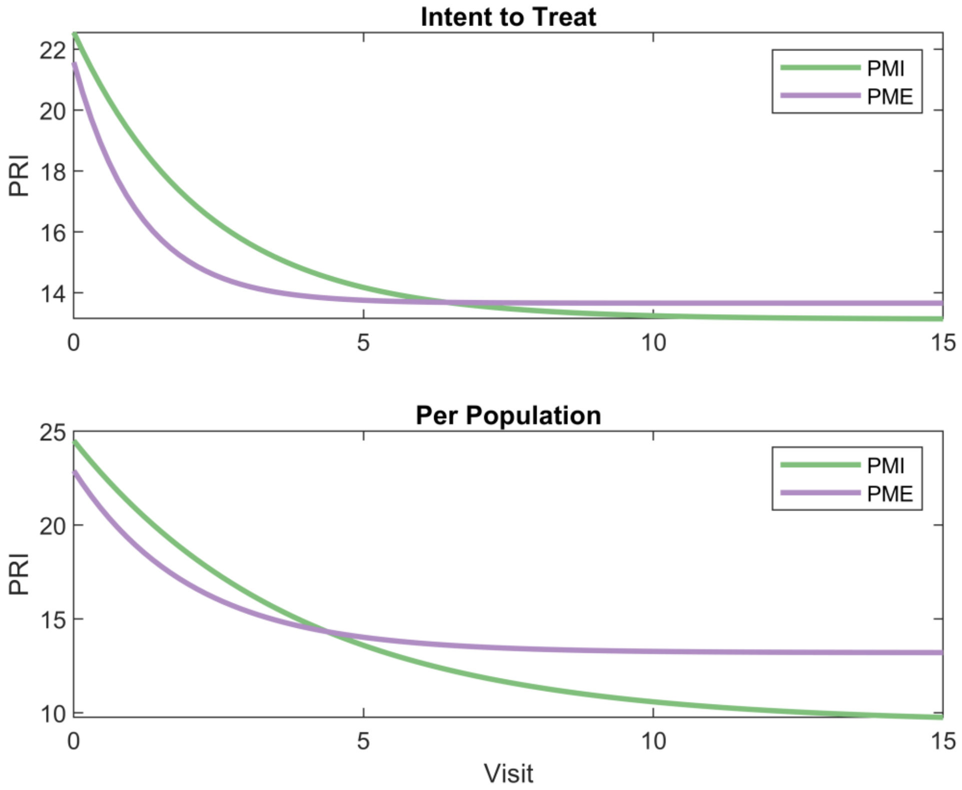

Figure S15: Exponential Decay model.

PRI scores along the entire treatment period have been model to fit an exponential decay for both the ITT (upper subplot) and PP populations (lower subplot). It is possible to see how the group receiving the experimental treatment (PME) has a steeper pain decrease in the earlier phases of the trial. Abbreviations: ITT, Intent-to-Treat; PP, Per Protocol; PME, Phantom Motor Execution; PMI, Phantom Motor Imagery; PRI, Pain Rating Index

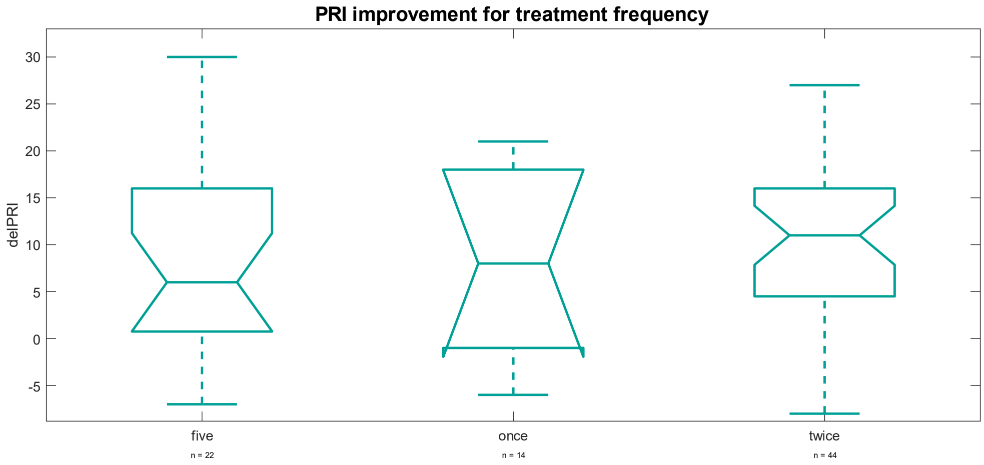

Figure S16: Impact of Treatment Frequency on Primary Outcome (ITT population)

This subplot visualizes the main outcome (delPRI) which is PRI difference between baseline (Visit 0) and end of treatment (Visit 15) of patients according to the frequency of their treatment sessions. The data illustrate that despite variations in treatment timing and session frequency, chosen post-randomization, there are no significant differences in patient response intensity (PRI) among the subgroups. This supports our hypothesis that the cumulative exposure to therapy, rather than specific session timings or frequency, is crucial for therapeutic effectiveness.

Abbreviations: ITT, Intent-to-Treat; PP, Per Protocol; PME, Phantom Motor Execution; PMI, Phantom Motor Imagery; delPRI, delta Pain Rating Index (difference between Visit 0 and Visist 15).

References

Andraszewicz, S., Scheibehenne, B., Rieskamp, J., Grasman, R., Verhagen, J., & Wagenmakers, E. J. (2015). An Introduction to Bayesian Hypothesis Testing for Management Research. *Journal of Management*, *41*(2), 521–543. https://doi.org/10.1177/0149206314560412

Anekar, A. A., & Cascella, M. (2022). WHO Analgesic Ladder. In *StatPearls*. http://www.ncbi.nlm.nih.gov/pubmed/32119322

Attal, N., Cruccu, G., Baron, R., Haanpää, M., Hansson, P., Jensen, T. S., & Nurmikko, T. (2010). EFNS guidelines on the pharmacological treatment of neuropathic pain: 2010 revision. *European Journal of Neurology*, *17*(9), 1113-e88. https://doi.org/10.1111/j.1468-1331.2010.02999.x

Collet, C., Guillot, A., Lebon, F., MacIntyre, T., & Moran, A. (2011). Measuring motor imagery using psychometric, behavioral, and psychophysiological tools. *Exercise and Sport Sciences Reviews*, *39*(2), 85–92. https://doi.org/10.1097/JES.0b013e31820ac5e0

Hayes, A. F. (2020). Comparing Two Independent Groups. In *Statistical Methods for Communication Science* (pp. 210–243). Routledge. https://doi.org/10.4324/9781410613707-10

Herdman, M., Gudex, C., Lloyd, A., Janssen, M., Kind, P., Parkin, D., Bonsel, G., & Badia, X. (2011). Development and preliminary testing of the new five-level version of EQ-5D (EQ-5D-5L). *Quality of Life Research*, *20*(10), 1727–1736. https://doi.org/10.1007/s11136-011-9903-x

Hobbins, A., Barry, L., Kelleher, D., Shah, K., Devlin, N., Goni, J. M. R., & O'Neill, C. (2018). Utility Values for Health States in Ireland: A Value Set for the EQ-5D-5L. *PharmacoEconomics*, *36*(11), 1345–1353. https://doi.org/10.1007/s40273-018-0690-x

Hurst, H., & Bolton, J. (2004). Assessing the clinical significance of change scores recorded on subjective outcome measures. *Journal of Manipulative and Physiological Therapeutics*, *27*(1), 26–35. https://doi.org/10.1016/j.jmpt.2003.11.003

Jensen, C. E., Sørensen, S. S., Gudex, C., Jensen, M. B., Pedersen, K. M., & Ehlers, L. H. (2021). The Danish EQ-5D-5L Value Set: A Hybrid Model Using cTTO and DCE Data. *Applied Health Economics and Health Policy*, *19*(4), 579–591. https://doi.org/10.1007/s40258-021-00639-3

Jones, S. M., Lange, J., Turner, J., Cherkin, D., Ritenbaugh, C., Hsu, C., Berthoud, H., & Sherman, K. (2016). *Development and Validation of the EXPECT Questionnaire : Assessing Patient Expectations of Outcomes of Complementary and Alternative Medicine Treatments for Chronic Pain*. *0*(0), 1–11. https://doi.org/10.1089/acm.2016.0242

Krekelberg, B. (2022). *bayesFactor* (1.0.0). GitHub. https://github.com/klabhub/bayesFactor

Kroenke, K., Spitzer, R. L., & Williams, J. B. W. (2001). The PHQ-9: Validity of a brief depression severity measure. *Journal of General Internal Medicine*, *16*(9), 606–613. https://doi.org/10.1046/j.1525-1497.2001.016009606.x

Lendaro, E., Hermansson, L., Burger, H., Van der Sluis, C. K., McGuire, B. E., Pilch, M., Bunketorp-Käll, L., Kulbacka-Ortiz, K., Rignér, I., Stockselius, A., Gudmundson, L., Widehammar, C., Hill, W., Geers, S., & Ortiz-Catalan, M. (2018). Phantom motor execution as a treatment for phantom limb pain: protocol of an international, double-blind, randomised controlled clinical trial. *BMJ Open*, *8*(7), e021039. https://doi.org/10.1136/bmjopen-2017-021039

Lendaro, E., Mastinu, E., Håkansson, B., & Ortiz-Catalan, M. (2017). Real-time Classification of Non-Weight Bearing Lower-Limb Movements Using EMG to Facilitate Phantom Motor Execution: Engineering and Case Study Application on Phantom Limb Pain. *Frontiers in Neurology*, *8*(SEP), 1–12. https://doi.org/10.3389/fneur.2017.00470

Ludwig, K., Graf von der Schulenburg, J. M., & Greiner, W. (2018). German Value Set for the EQ-5D-5L. *PharmacoEconomics*, *36*(6), 663–674. https://doi.org/10.1007/s40273-018-0615-8

Makin, T. R., & Orban de Xivry, J.-J. (2019). Ten common statistical mistakes to watch out for when writing or reviewing a manuscript. *ELife*, *8*. https://doi.org/10.7554/eLife.48175

Melzack, R. (1975). The McGill Pain Questionnaire: Major properties and scoring methods. *Pain*, *1*(3), 277–299. https://doi.org/10.1016/0304-3959(75)90044-5

Melzack, R. (1987). The short-form McGill pain questionnaire. *Pain*, *30*(2), 191–197. https://doi.org/10.1016/0304-3959(87)91074-8

Mooney, T. K., Beth, M., Gibbons, C., Gallop, R., Mack, R. A., & Crits-christoph, P. (2015). *Credibility and the Relation of Credibility to Therapy Outcome*. *24*(5), 565–577. https://doi.org/10.1080/10503307.2013.847988.Psychotherapy

Morey, R. D., Romeijn, J. W., & Rouder, J. N. (2016). The philosophy of Bayes factors and the quantification of statistical evidence. *Journal of Mathematical Psychology*, *72*, 6–18. https://doi.org/10.1016/j.jmp.2015.11.001

Nicholas, M. K. (2007). The pain self-efficacy questionnaire: Taking pain into account. *European Journal of Pain*, *11*(2), 153–163. https://doi.org/10.1016/j.ejpain.2005.12.008

Ortiz-Catalan, M., Gudmundsdottir, R. A., Kristoffersen, M. B. M. B., Zepeda-Echavarria, A., Caine-Winterberger, K., Kulbacka-Ortiz, K., Widehammar, C., Eriksson, K., Stockselius, A., Ragnö, C., Pihlar, Z., Burger, H., Hermansson, L., Guðmundsdóttir, R. A. R. A., Kristoffersen, M. B. M. B., Zepeda-Echavarria, A., Caine-Winterberger, K., Kulbacka-Ortiz, K., Widehammar, C., … Hermansson, L. (2016). Phantom motor execution facilitated by machine learning and augmented reality as treatment for Phantom Limb Pain. *The Lancet*, *388*(10062), 2885–2894. https://doi.org/10.1016/S0140-6736(16)31598-7

Ortiz-Catalan, M., Sander, N., Kristoffersen, M. B., Håkansson, B., & Brånemark, R. (2014). Treatment of phantom limb pain (PLP) based on augmented reality and gaming controlled by myoelectric pattern recognition: A case study of a chronic PLP patient. *Frontiers in Neuroscience*. https://doi.org/10.3389/fnins.2014.00024

Pickard, A. S., Law, E. H., Jiang, R., Pullenayegum, E., Shaw, J. W., Xie, F., Oppe, M., Boye, K. S.,

Chapman, R. H., Gong, C. L., Balch, A., & Busschbach, J. J. V. (2019). United States Valuation of EQ-5D-5L Health States Using an International Protocol. *Value in Health*, *22*(8), 931–941. https://doi.org/10.1016/j.jval.2019.02.009

Rencz, F., Brodszky, V., Gulácsi, L., Golicki, D., Ruzsa, G., Pickard, A. S., Law, E. H., & Péntek, M. (2020). Parallel Valuation of the EQ-5D-3L and EQ-5D-5L by Time Trade-Off in Hungary. *Value in Health*, *23*(9), 1235–1245. https://doi.org/10.1016/j.jval.2020.03.019

Sullivan, M. J. L., Bishop, S. R., & Pivik, J. (1995). The Pain Catastrophizing Scale: Development and validation. *Psychological Assessment*, *7*(4), 524–532. https://doi.org/10.1037/1040-3590.7.4.524

Tait, R. C., Chibnall, J. T., & Krause, S. (1990). The Pain Disability Index: psychometric properties. *Pain*, *40*(2), 171–182. http://www.ncbi.nlm.nih.gov/pubmed/2308763

Versteegh, M., M. Vermeulen, K., M. A. A. Evers, S., de Wit, G. A., Prenger, R., & A. Stolk, E. (2016). Dutch Tariff for the Five-Level Version of EQ-5D. *Value in Health*, *19*(4), 343–352. https://doi.org/10.1016/j.jval.2016.01.003

Williams, G. C., Freedman, Z. R., & Deci, E. L. (1998). Supporting autonomy to motivate patients with diabetes for glucose control. *Diabetes Care*, *21*(10), 1644–1651. https://doi.org/10.2337/diacare.21.10.1644

World Health Organization. (2018). WHO guidelines for the pharmacological and radiotherapeutic management of cancer pain in adults and adolescents. In *World Health Organization*.

Xie, F., Pullenayegum, E., Gaebel, K., Bansback, N., Bryan, S., Ohinmaa, A., Poissant, L., & Johnson, J. A. (2016). A Time Trade-off-derived Value Set of the EQ-5D-5L for Canada. *Medical Care*, *54*(1), 98–105. https://doi.org/10.1097/MLR.0000000000000447
